# Association between hydrometeorological conditions and hemorrhagic fever with renal syndrome in Shandong Province, China, from 2005 to 2019

**DOI:** 10.1371/journal.pntd.0013306

**Published:** 2025-07-24

**Authors:** Qi Gao, Chuanlong Cheng, Hui Zuo, Rui Xi, Zhiqiang Wang, Xiujun Li

**Affiliations:** 1 Department of Biostatistics, School of Public Health, Cheeloo College of Medicine, Shandong University, Jinan, Shandong, China; 2 Institute of Infectious Disease Control and Prevention, Shandong Center for Disease Control and Prevention, Jinan, Shandong, China; National Institute for Communicable Disease Control and Prevention, China CDC, CHINA

## Abstract

**Background:**

Hemorrhagic fever with renal syndrome (HFRS) is a rodent-borne zoonotic disease with a case fatality rate ranging from 1% to 15%. Long-term evidence regarding its association with local hydrometeorological conditions remain limited. This study aimed to assess the non-linear and lagged effects of extreme hydrometeorological conditions on HFRS risk and examine the modifying effects of regional characteristics in Shandong Province, China.

**Methods:**

Data from January 1, 2005 to December 31, 2019 across 136 counties in Shandong Province were collected. The Standardized Precipitation Evapotranspiration Index (SPEI), calculated from temperature, precipitation and evaporation, was used to represent local hydrometeorological conditions (dry and wet). A spatiotemporal Bayesian hierarchical model combined with distributed lag non-linear model was applied to explore the association between climate indicators and HFRS risk over a 6-month lag. Modification effects were quantified using linear interaction terms.

**Results:**

Over the 15-year period, annual HFRS incidence declined from 2.62/100,000 to 0.72/100,000, with two minor peaks observed. The cumulative association between SPEI and HFRS over 6-month lag appeared U-shaped. The relative risk (RR) of HFRS under extreme wet conditions increased at 4–6 months lag, peaking at the 6-month lag (RR = 1.49, 95% confidence interval (CI): 1.37-1.63). Extreme dry conditions had a persistent impact, also peaking at the 6-month lag (RR = 1.05, 95% CI: 1.01-1.09). Areas with low per capita Gross Domestic Product, Normalized difference vegetation index, Total power of agricultural machinery and annual temperature, as well as high elevation, exhibited higher risks of HFRS under extreme wet conditions. The modification effects under extreme dry conditions were similar but weaker.

**Conclusions:**

Both extreme wet and dry conditions increase the risk of HFRS, with county characteristics further modifying these associations. These findings provide a scientific foundation for policymakers to develop targeted and effective HFRS prevention and control strategies, particularly in high-risk regions, while considering hydrometeorological conditions.

## Author summary

Hemorrhagic fever with renal syndrome (HFRS) is a widespread zoonotic disease caused by rodent-transmitted Hantaviruses. Although prevention efforts in China have reduced HFRS incidence, the disease remains a significant threat in regions such as Shandong Province, especially with the challenges posed by climate change. In this study, we used a spatiotemporal Bayesian hierarchical model combined with distributed lag nonlinear model to assess the nonlinear and lagged effects of hydrometeorological conditions on HFRS incidence, and explore the modifying effects of regional characteristics in these associations. The results of this study revealed a U-shaped cumulative association, with both wet and dry conditions associated with increased HFRS within a 6-month lag period. County-level characteristics, such as economic, environmental, and climatic factors significantly modified these associations. The relative risks under extreme wet conditions were higher in area with low per capita Gross Domestic Product, Normalized difference vegetation index, Total power of agricultural machinery and annual temperature, as well as areas with high elevations. The modification effects under extreme dry conditions were similar but less pronounced. These findings provide valuable guidance for developing climate change adaptation strategies and targeted public health interventions to reduce the risk of HFRS outbreaks, particularly in vulnerable regions.

## Introduction

Hemorrhagic fever with renal syndrome (HFRS) is a global rodent-borne zoonotic infectious disease caused by Hantaviruses (HV), with the case fatality rate ranging from 1%–15% [1]. This disease has been reported over 70 countries, primarily in Asia and Europe [2,3], with approximately 100, 000 new cases annually [4]. China is the most prevalent country, accounting for 70%-90% of global cases (576,361 cases reported between 1995 and 2020) [3,4]. Control measures in China, such as rodent management, improved environments and vaccination, have reduced HFRS incidence [2]. However, HFRS remains a significant public health concern in certain regions, such as Shandong Province [5]. Historically, Shandong has been a high burden region, reporting its first case in 1962 and accounting for about one-third of national cases [2]. Even during the COVID-19 pandemic in 2021, the disease continued to spread, with 875 cases reported [6].

Climate change, a major global challenge, threatens human health by altering temperature and precipitation patterns [7]. These changes can affect local hydrometeorological conditions (i.e., the combination of local temperature, precipitation and evaporation) and impact infectious disease dynamics, especially for vector-borne infectious diseases [8,9]. Previous researches indicated that climate dynamics strongly influence the magnitude and seasonality of HFRS transmission, with temperature and precipitation being key determinants of its epidemiological patterns [10–13]. As a rodent-borne disease, HFRS transmission is particularly sensitive to hydrometeorological events such as droughts, floods and tropical cyclones, which can alter rodent habitats and human activity [14–18]. For example, extreme precipitation has been shown to increase HFRS risk within a six-month period [19]. The effects of climate variability on HFRS are complex, often lagged, and evidence regarding nonlinear and lagged effects of hydrometeorological events is still limited.

As a zoonotic infectious disease, the transmission and prevalence of HFRS is shaped by demographic, geographic, and seasonal factors. Local hydrometeorological conditions interact with regional characteristics, influencing rodent ecology and human exposure risks [5,20,21]. Urbanization, population density, and prevention measures contribute to regional differences in HFRS incidence. For example, a previous study indicated that HFRS cases were significantly associated with local climatic factors, normalized difference vegetation index (NDVI), and socioeconomic factors in preceding months [11]. Another long-term study revealed a biphasic inverted U-shaped relationship between the HFRS incidence and urbanization [22]. These findings suggest that regional characteristics may modify the effects of hydrometeorological conditions on HFRS incidence. Fully exploring the multidimensional effects of regional characteristics is essential for developing targeted adaptation strategies and effective early warning systems, particularly in the context of global climate change and accelerating urbanization.

To address these challenges, we applied a county-level spatiotemporal Bayesian hierarchical model integrated with a distributed lag nonlinear model (DLNM). This framework allows for comprehensive assessment of the exposure-response relationship by simultaneously accounting for spatial heterogeneity, temporal dynamics, and lagged effects of exposure. Using 15 years of HFRS surveillance data from Shandong Province, we quantified the nonlinear and lagged associations between hydrometeorological conditions and HFRS incidence, while also assessing the modification effects of regional characteristics. These findings will help reveal HFRS epidemic patterns and provide a scientific basis for developing more targeted prevention and control strategies.

## Materials and methods

### Study area

**Fig 1:**
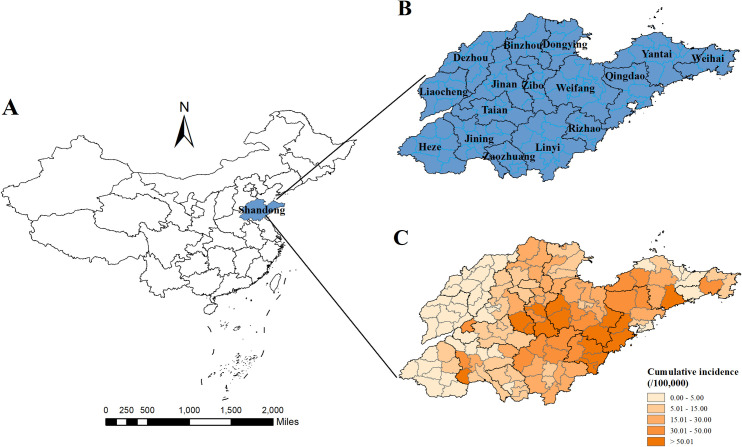
Geographic locations and spatial distribution of cumulative HFRS incidence for the 136 counties of the Shandong Provinces in China From 2005 to 2019. The base map is from the data center for geographic sciences and natural sources research, CAS (http://www.resdc.cn/data.aspx?DATAID=201).

Shandong Province (latitude 34°23′-38°17′N and longitude 114°48′-122°42′E) is located on the eastern coast of China. By the end of 2019, it covered an area of 157,965 km^2^ and had a population of 100.7 million, making it the second most populous province in China (http://tjj.shandong.gov.cn/). Administratively, Shandong is divided into 16 prefecture-level cities, which contain 136 county-level administrative units: 58 municipal districts, 26 county-level cities, and 52 counties (Fig 1). The province experiences a warm temperate continental monsoon climate, with average temperatures ranging from -10.21°C to 31.76°C.

### Data collection

Daily HFRS cases data from January 2005 to December 2019 at county level were obtained from the the National Notifiable Diseases Surveillance System (NNDSS) of China Center for Disease Control and Prevention (CDC) [3]. This internet-based, real-time reporting system covers all national notifiable diseases. In China, HFRS is classified as a category B infectious disease, requiring mandatory online reporting to NNDSS within 24 hours after diagnosis. All HFRS cases included in this study were diagnosed according to the national protocol (WS 278–2008), ensuring consistency across regions and years. Only clinically and laboratory-confirmed cases were included, and suspected cases were excluded. The dataset contained information on onset date, sex, age, and residential administrative codes, with all personally identifiable information was removed to ensure confidentiality. Data cleaning and quality control were performed, including validation of demographic information, outlier detection (such as large differences between onset date and diagnosis date), and removing duplicate records. To reduce excess zeros and improve data robustness, the daily case data were aggregated to monthly counts at the county level [23].

Meteorological variables, including mean temperature, relative humidity, and cumulative precipitation for the same period, were obtained from the land component of the fifth generation of the European reanalysis (ERA5-land) dataset. ERA5-Land data was selected for its high spatial and temporal resolution (0.1° × 0.1°), long-term coverage, and relatively good performance [24]. Local hydrometeorological conditions were quantified using the Standardized Precipitation Evapotranspiration Index (SPEI), which describes a range of dry and wet conditions [25]. Following previous study, SPEI values above 2 indicated extreme wet conditions, and values below -2 indicated extreme dry conditions [26]. SPEI was calculated at 1-, 3- and 6-month scales to represent monthly, seasonal and medium-term hydrometeorological conditions, respectively [27].

Based on biological relevance to HV transmission and data availability, sixteen variables were initially considered (S1 Table and S1 Fig). Pairwise correlations were conducted to reduce collinearity and highly correlated variables were removed (S2 Fig). Six county level annual variables were finally selected (S2 Table). Among these, per capita Gross Domestic Product (GDP) and total power of agricultural machinery (TPAM) from 2005 to 2019 were extracted from the Shandong Statistical Yearbook (http://tjj.shandong.gov.cn/). Missing values were imputed using the k-nearest neighbor (KNN) method [28]. Nighttime light data were obtained from the National Tibetan Plateau Data Center (http://data.tpdc.ac.cn/) [29]. Population density (https://www.worldpop.org/), NDVI, elevation (https://www.resdc.cn/) and annual land cover data [30] were also collected from public available raster sources. All raster data were summarized at the county-level using a vector map of Shandong Province, and calculated via the ‘Zonal Statistics Tool’ in ArcGIS 10.8.

### Statistical analysis

A spatiotemporal Bayesian hierarchical model was constructed to examine the association between SPEI and HFRS in 136 counties from January 2005 to December 2019. The model incorporated spatiotemporal random effects to account for unobserved and unmeasured variability, as well as spatial and temporal dependence structures. To account for overdispersion in HFRS counts, a negative binomial distribution was employed, which provides a flexible approach to variance inflation in epidemiological count data [31]. The model was specified as follows:


$$Y_{it}\sim NegBin\left(\mu_{it},\kappa \right)$$

$$\log{(\mu }_{it})=\log{(pop}_{it})+\log{(p}_{it})$$

where$\;Y_{it}$ is the count of HFRS cases in country *i* (*i* = 1, 2, …,136) during month *t*, $\mu_{it}$ is the corresponding mean of distribution, and κ is the overdispersion parameter. $\mu_{it}$ was calculated as the product of the county population per 100,000 $\left({pop}_{it}\right)$ and undiscovered HFRS incidence rate $p_{it}$ [32]. Population effects were modelled by including the log of the population as an offset.

The model was extended with a distributed lag nonlinear model (DLNM) to quantify the lagged and nonlinear association between climate indicators and HFRS incidence as follows:


$$\log\left(\mu_{it}\right)=\alpha +\beta_{s(i)m(t)}+\upsilon_{ia(t)}+\varphi_{ia(t)}+\delta_{it}+cb(SPEI,l)+cb(met,l)$$

where α is the intercept, $\beta_{s(i)m(t)}$ represents the random seasonal effect of the month m(t) for city *i* to account for seasonal autocorrelation, specified as a cyclic first-order random walk [32]. $\upsilon_{ia(t)}\;and\;\varphi_{ia(t)}$ denote structured and unstructured spatial random effects at the county-level, jointly specified by the Besag-York-Mollié2 (BYM2) model. $\delta_{it}$ is a space-time interaction term to capture residual spatiotemporal variation. cb(SPEI,l) and cb(met,l) represent the cross-basis functions modeling nonlinear lagged effects of hydrometeorological conditions, temperature, humidity and precipitation, using natural cubic splines with 3 degrees of freedom (*df*). Given the incubation period of HFRS and the delayed transport of pathogens in the rodent and external environment, lag periods was set at 0–6 months to explore all possible lagged associations [18,33]. A Penalized Complexity (PC) prior was applied for the precision parameter $\tau =1/\sigma^{2}$, with Pr ($1/\sqrt{}\tau >0.5$) = 0.01 [34]. Model parameters were estimated in a Bayesian framework using the Integrated Nested Laplace Approximation (INLA) method.

A stepwise modeling strategy was applied to estimate the association between county-level HFRS incidence and climate indicators with varying adjustments for county characteristics (S3 Table). First, a basic model including only monthly random effects, without adjustment for county-specific characteristics was fitted. Second, spatial random effects were added, followed by the inclusion of a space-time interaction term. Third, we introduced SPEI at different time scales (1-, 3-, and 6-month) and evaluated model performance using the deviance information criterion (DIC) and mean cross-validated log score, with lower values indicating better fit. The model incorporating SPEI-6 was selected as the best-fitting model. Subsequently, meteorological variables were included to examine potential confounding. Finally, to explore the modification effects of county-level characteristics, a linear interaction term between cb(SPEI−6,lag) and each county indicator was included. Each indicator was centered on the 10^th^, 50^th^ and 90^th^ percentiles of the 136 counties to assess the effect of SPEI-6 at different levels of county development.

Sensitivity analyses were performed to test the robustness of the model. These included 1): changing the *df* (2–4) for SPEI-6 in cross-basis matrices, 2) changing the *df* (3–5) for SPEI-6 lag in cross-basis matrices, 3) altering the combination terms of hydrometeorological and meteorological factors, 4) stratifying the data into autumn-winter and spring seasons based on predominant infection periods of different hantavirus genotypes, 5) using leave-one-out cross-validation by repeatedly excluding one month per year, generating out-of-sample posterior predictive distributions.

The spatiotemporal model was implemented using “dlnm” [35] and “INLA” [36] packages in R version 4.2.3.

## Results

### Descriptive analysis

**Fig 2:**
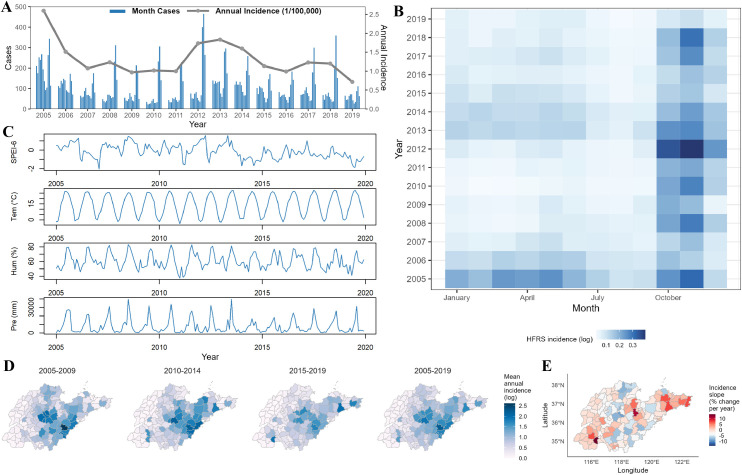
Temporal and geographical trends of HFRS cases and meteorological factors in Shandong, 2005–2019. (A) Monthly HFRS cases and annual incidence. (B) Seasonal distribution of monthly HFRS incidence. (C) Time series of meteorological factors at the provincial level. (D) Geographical distribution of mean annual HFRS incidence for each 5-year period. (E) Estimated slopes of annual HFRS incidence from 2005 to 2019 (% change per year), showing counties with increasing (red) or decreasing (blue) trends. The base map is from the data center for geographic sciences and natural sources research, CAS (http://www.resdc.cn/data.aspx?DATAID=201).

From 2005 to 2019, a total of 19,121 HFRS cases and 249 deaths were reported in 136 counties across Shandong Province. Among the reported cases, 72.40% were male, 49.14% were aged 40–60 years, and 82.45% were farmers (Table 1). During this 15-year period, the annual HFRS incidence decreased from 2.62/100,000 to 0.72/100,000, with two minor peaks observed (Fig 2A). The disease showed a clear bimodal seasonal pattern, with a major peak in autumn and winter (October to January) and a minor peak in spring (March to June) (Figs 2B and S3). The interval between the spring and autumn-winter peaks fluctuated over time. Hydrometeorological conditions and meteorological variables in Shandong Province also exhibited seasonal fluctuations during the study period (Figs 2C, S4 and S5).

**Table 1:** Characteristics of HFRS cases of Shandong province in China, 2005–2019.

Characteristic	Cases, No. (%)	Death, No. (%)
All	19,102 (100.00)	249 (100.00)
Gender		
Male	13,829 (72.40)	186 (74.70)
Female	5,273 (27.60)	63 (25.30)
Age (years)		
< 20	789 (4.13)	1 (0.40)
20-39	4,823 (25.25)	29 (11.65)
40-60	9,387 (49.14)	139 (55.82)
60-80	3,849 (20.15)	73 (29.32)
≥ 80	254 (1.33)	7 (2.81)
Occupation		
Farmer	15,749 (82.45)	203 (81.53)
Worker	1,845 (9.66)	26 (10.44)
Student	588 (3.08)	1 (0.40)
Others	920 (4.82)	19 (7.63)

There were significant geographical variations in HFRS incidence, with incidence ranging from 0 to 18.71 per 100,000 population (S6 Fig). Over time, high-incidence areas become more localized and concentrated, mainly in the central and southeastern regions, with sporadic outbreaks occurring in the southwestern and northeastern peninsular regions (Fig 2D). Linear regression was employed to estimate the annual log incidence and map its directional trends at county-level (Fig 2E). The slopes indicated a declining trend in the most counties, while several counties in the southwestern, central and northeastern peninsular regions exhibited a significant (*P *< 0.05) upward trend, with some counties experiencing increases up to a 14%.

### Association between climate indicators and HFRS

**Fig 3:**
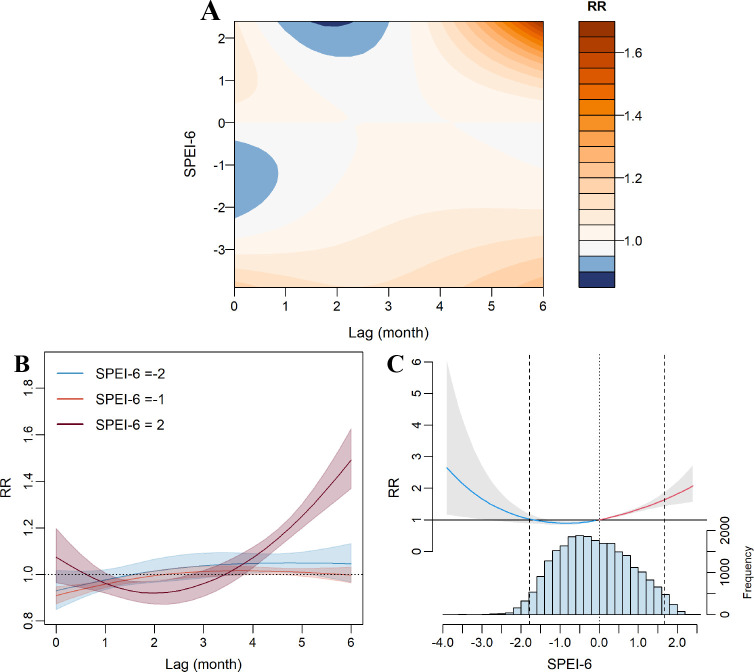
Exposure-lag-response associations between SPEI-6 and HFRS. (A) Association between HFRS risk and SPEI-6 at different exposure and lag month. The color gradient represents RR values, with warmer colors indicating higher risk. (B) Lag–response association of HFRS risk at SPEI-6 values of -2, -1 and 2. Solid lines indicate the estimated effects, and shaded areas represent 95% CI. (C) Cumulative exposure-response association between SPEI-6 and HFRS. The solid vertical line denotes the central values of SPEI-6, and dashed vertical lines represent the 2.5^th^ and 97.5^th^ percentiles.

Fig 3 presents the impact of SPEI-6 on HFRS incidence. The contour plot shows that the association between SPEI-6 and HFRS incidence is nonlinear and displays a lagged effect. Compared to normal hydrometeorological conditions, wet conditions have a noticeable impact both in the early stages and after 4 month lag. Under wet conditions, the early risk is relatively mild, but the effect intensifies over time and peaks at the 6-month lag (RR = 1.49, 95% confidence interval (CI): 1.37-1.63) (Fig 3A). Slice effect plots further demonstrate the trend of this effect with varying lag periods. In contrast, extreme dry conditions show a persistent but smaller impact throughout the lag period, peaking at 6 month (RR = 1.05, 95% CI: 1.01-1.09). The cumulative association between SPEI and HFRS over a 6-month lag followed a U-shaped curve (Fig 3C). The cumulative RR was 1.10 (95% CI: 0.91-1.33) for extreme dry conditions (SPEI = -2) and 1.82 (95% CI: 1.50-2.21) for extreme wet conditions (SPEI = 2).

The shapes of the exposure, lag response, and cumulative curves varied across the meteorological variables (S7 Fig). For temperature, a nonlinear, L-shaped relationship was observed, with the highest cumulative RR at a monthly temperature of -5.4 °C (S7A Fig). For relative humidity, the association showed an opposite pattern, with the highest cumulative RR at 89% (S7B Fig). HFRS risk increased significantly when monthly cumulative precipitation exceeded the median value of 29 mm (S7C Fig). Subgroup analyses by gender and occupation showed similar associations between hydrometeorological conditions and HFRS incidence (Table 2 and S8 Fig). The effect of extreme wet conditions was more pronounced in individuals aged < 20 years old (RR = 1.85, 95% CI: 1.42-2.42). Sensitivity analyses indicated that the model results were robust when adjusting the *df* for exposure and lag space, and meteorological variables in the model (S9A-G Fig). When stratified by season, the association between SPEI-6 and HFRS incidence differed between autumn-winter and spring (S9H-I Fig). In autumn-winter, both extreme dry and wet conditions were associated with higher risk, showing a U-shaped relationship. In contrast, the association in spring was weaker and not statistically significant. Cross-validation results indicated that the predicted cases closely matched the reported cases and successfully captured the epidemic trend (S10 Fig).

**Table 2:** Maximum and cumulative risk of HFRS under extreme wet and extreme dry conditions within 6 months lag, stratified by gender, age and occupation.

	Extreme wet (SPEI = 2)			Extreme dry (SPEI = -2)		
	Maximum RR (95% CI)	Lag, month	Cumulative RR (95% CI)	Maximum RR (95% CI)	Lag, month	Cumulative RR (95% CI)
Overall	1.49 (1.37,1.63)	6	1.82 (1.50,2.21)	1.05 (1.01,1.09)	5	1.10 (0.91,1.33)
Gender						
Male	1.52 (1.39, 1.66)	6	1.98 (1.61, 2.44)	1.05 (1.01, 1.10)	4	1.13 (0.92, 1.39)
Female	1.46 (1.29, 1.67)	6	1.31 (0.98, 1.77)	1.08 (1.00, 1.16)	5	1.41 (1.08, 1.84)
Age (years)						
< 20	1.85 (1.42, 2.42)	6	3.19 (1.77, 5.76)	1.13 (0.92, 1.38)	6	1.65 (1.05, 2.60)
20-39	1.58 (1.39, 1.80)	6	2.13 (1.59, 2.83)	1.11 (1.04, 1.20)	3	1.34 (1.01, 1.76)
40-60	1.49 (1.34, 1.66)	6	1.62 (1.27, 2.06)	1.07 (0.97, 1.18)	6	1.22 (0.97, 1.54)
60-80	1.32 (1.13, 1.54)	6	1.10 (0.77, 1.56)	1.16 (1.03, 1.31)	6	1.44 (1.08, 1.92)
≥ 80	1.20 (0.70, 2.06)	0	0.92 (0.30, 2.75)	1.19 (0.88, 1.62)	0	2.16 (1.18, 3.94)
Occupation						
Farmer	1.45 (1.33, 1.60)	6	1.74 (1.42, 2.15)	1.05 (1.01, 1.10)	5	1.11 (0.90, 1.36)
Worker	1.78 (1.48, 2.14)	6	2.80 (1.84, 4.27)	1.13 (1.05, 1.21)	4	1.81 (1.28, 2.54)
Student	1.81 (1.35, 2.44)	6	2.79 (1.42, 5.47)	1.15 (0.92, 1.43)	6	1.64 (1.01, 2.67)
Others	1.48 (1.12, 1.94)	6	0.94 (0.49, 1.79)	1.25 (1.05, 1.50)	6	2.39 (1.60, 3.56)

### Modification role of county characteristics

**Fig 4:**
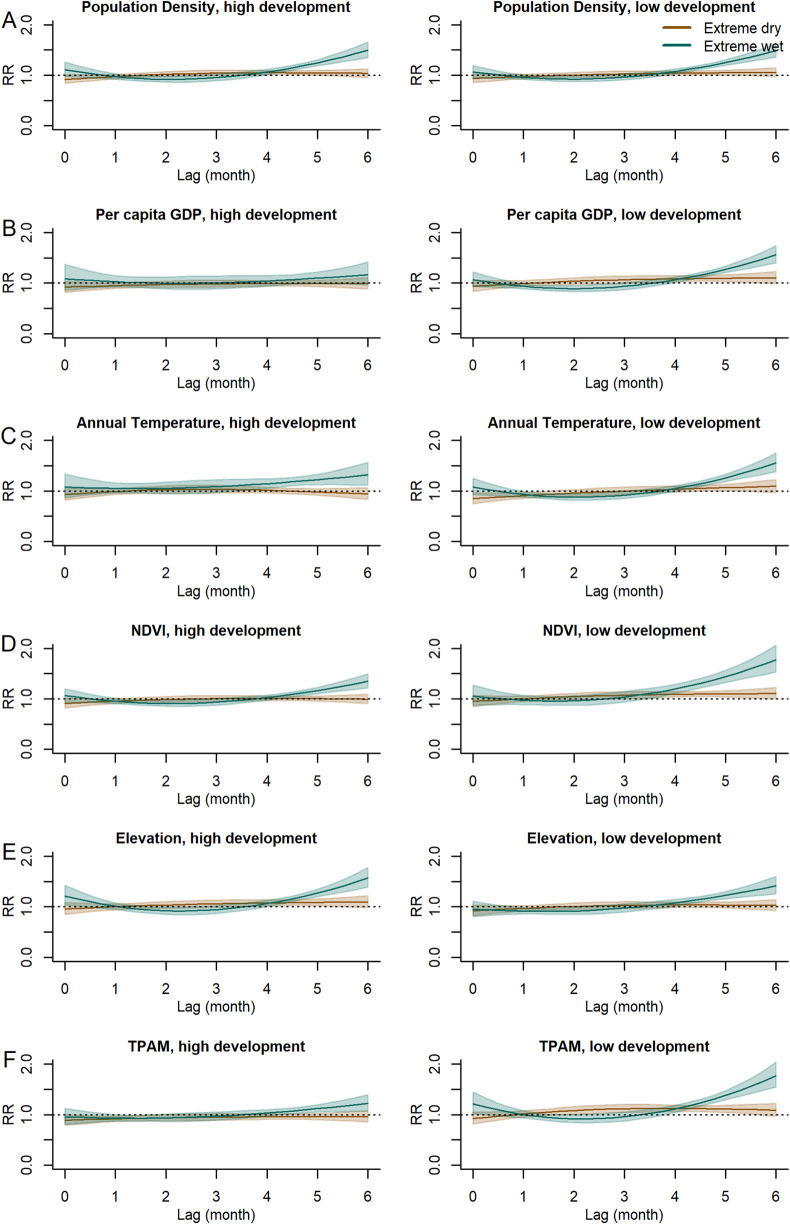
Modification effects of county characteristics on the association between SPEI-6 and HFRS. (A–F) Scenarios with a high and low levels of county characteristics were demonstrated, respectively). For each interaction, the county characteristics was centered on its 10^th^ and 90^th^ percentiles of the values observed among the 136 counties.

Based on variable correlations, six county-level indicators were selected in the interactive analyses: population density, per capita GDP, annual temperature, NDVI, elevation and TPAM (S2 Fig). These variables represent different regional characteristics, including economic status, ecological environment, and other aspects. The results showed that county characteristics significantly modified the association between hydrometeorological conditions and HFRS risk (Figs 4, S11 and S4 Table). For example, in counties with low per capita GDP, annual temperature, NDVI and TPAM, the risk of HFRS under extreme wet conditions increased substantially, peaking at a 6-month lag: low per capita GDP (RR = 1.56, 95% CI: 1.40-1.74), annual temperature (RR = 1.55, 95% CI: 1.38-1.55), NDVI (RR = 1.78, 95% CI: 1.53-2.06), and TPAM (RR = 1.77, 95% CI: 1.54-2.04). The modification effects under extreme dry conditions were generally weaker than those observed for extreme wet conditions. In high-value areas for these indicators, extreme dry conditions showed no significant effects, as observed in counties with high GDP (RR = 0.99, 95% CI: 0.94-1.04) and high TPAM (RR = 0.96, 95% CI: 0.88-1.05).

Under extreme wet or dry conditions, population density did not show a significant modifying effect, though a slight increase in cumulative RR was observed in extreme wet environments (RR = 1.86, 95% CI: 1.48-2.35). With respect to geographical factors, areas with high elevation were more susceptible to increased HFRS risk under extreme wet conditions. The risk of HFRS increased 4–6 months after extreme wet conditions, with the maximum single-month RR was 1.57 (95% CI: 1.39-1.77) at a 6-month lag and the cumulative RR were 2.25 (95% CI: 1.69-2.98). The modification effects under extreme dry conditions were similar, with only cumulative RR was significant (RR = 1.34, 95% CI: 1.01-1.77).

## Discussion

Given the serious public health threat posed by HFRS, it is essential to enhance our understanding of its epidemiological characteristics, key risk factors and potential modifying factors to develop targeted prevention and control strategies. This study examined the nonlinear and lagged associations between hydrometeorological conditions and HFRS incidence, and further explored the modifying effects of multidimensional socioeconomic and environmental indicators. We found that both extreme wet and dry conditions increased the risk of HFRS, with wet conditions having a stronger effect. The risk associated with dry conditions persisted throughout the lag period, while risk during extreme wet conditions persisted at a 4–6 month lag. Effects of hydrometeorological conditions did not differ significantly by gender or occupation, but individuals < 20 years old were more susceptible to extreme wet conditions. Additionally, county-level socioeconomic and environmental characteristics significantly modified these associations, with more pronounced effects under extreme wet conditions.

In this study, the increased risks of HFRS associated with extreme wet and dry conditions are consistent with previous studies on the effects of droughts and floods, respectively [14,17,37,38]. For example, a cross-sectional study demonstrated that severe floods were linked to an increased risk of HFRS over a three-year period [17]. Several plausible mechanisms may explain the elevated HFRS risk under extreme hydrological conditions. Humid environments promote the survival of rodents and enhance the stability and infectivity of hantavirus in ex-vivo environments [39]. Extreme wet environments can also alter rodent habitats, indirectly influencing HFRS transmission. For instance, after flooding, rodents may migrate to residential areas such as sewers and warehouses [37]. Such migration can increase rodent density over several generations breeding cycles, increasing the risk of virus transmission among rodents and sustaining heightened threats to nearby residents [40]. Factors such as overcrowded living conditions, poor sanitation, and limited access to healthcare may further facilitate virus spread [18]. In the short term, interventions such as rodent control, reduced crop yields and higher rodent mortality during flooding might explain the initial decreases in HFRS cases after extreme wet events [17,41]. However, increased rodent contact and delayed human infection due to incubation periods may result in more HFRS cases several months after periods of extreme humidity and heavy rainfall.

Compared to wet conditions, dry conditions were associated with a smaller but more prolonged increase risk of HFRS. Water scarcity during drought can affect agricultural production and alter seasonal work patterns, increasing human-rodent contact [42]. Previous studies have also reported a causal relationship between crop yields and HFRS incidence [21,43]. Food shortages caused by dry conditions may force rodents to seek sustenance in human settlements, increasing their reliance on human living environments and accelerating disease transmission. Our findings emphasize the impact of extreme weather events driven by climate change on HFRS epidemiology, underscoring the necessity of enhancing monitoring and tailored prevention strategies to address extreme hydrometeorological conditions.

Stratified analysis showed that individuals aged < 20 years old were more vulnerable to extreme wet conditions. This increased vulnerability may be attributed to their increased outdoor exposure, such as commuting to school during floods or heavy rainfall, which increases their susceptibility to infection [44]. In contrast, older adults (aged 60–80 years) are less likely to participate in outdoor activities due to physical limitations, reducing their exposure and risk. Additionally, there are no statistically significant differences in HFRS risk by gender or occupational. Evidence from other studies regarding gender and occupation-based vulnerability to climate factors has been inconsistent [44,45]. The lack of detailed individual behavioral data limits our ability to interpret these inconsistencies, further exploration is needed to better inform protection strategies for vulnerable populations [46].

Based on the correlation analysis results, six county-level indicators representing different regional characteristics were selected for the interactive analyses to avoid collinearity and ensure robust model estimates. Our results indicated that local economic levels and ecological environments significantly modified the relationship between hydrometeorological conditions and HFRS incidence. Areas with lower per capita GDP exhibited a higher risk during extreme wet conditions. Residents in economically disadvantaged areas may be less adapt to extreme wet conditions, possibly due to inadequate safety and health awareness, insufficient medical infrastructure, and limited public health services, which delay the identification and treatment of cases [47]. NDVI values represent the growth status and coverage of surface vegetation, with lower values indicating sparse vegetation or built environments, which may increase outdoor exposure and contact with rodents [48]. Low TPAM values reflect a reliance on manual agricultural labor with limited mechanization, further increasing exposure risk [49]. Given the critical role of animal hosts in the transmission process, host-targeted interventions and early monitoring of rodent population should be prioritized in high risks regions.

We also found that areas with low annual temperature had higher HFRS risk under extreme wet conditions. This may be because the reproductive rate of rodents is higher in colder environments, while reproduction can be inhibited when temperatures exceed a certain range [50,51]. Winter temperatures may also impact food supply and rodent overwinter survival, increasing rodent encroachment into human settlements [52,53]. The joint effects of low temperature and extreme wet events further amplify HFRS risk. Additionally, our results showed that higher elevation areas were more vulnerable to hydrometeorological extremes, consistent with prior findings [54]. In Shandong, high elevation areas are mainly mountainous and hilly, providing forest habitats that are favorable for rodents. This increases the likelihood of human contact with rodents and their excreta. These findings highlight the complex interaction between regional characteristics and hydrometeorological events in shaping HFRS incidence. Incorporating socioeconomic factors into models is essential for developing targeted public health strategies to address this evolving threat.

Several limitations of this study should be noted. First, Shandong is a mixed epidemic region where both HTNV and SEOV circulate. Due to limited data, we were unable to distinguish the genotypes of hantavirus in reported cases. Although we performed a sensitivity analysis using infection season as a proxy for virus type, this method is only as a rough surrogate and may not accurately reflect the true virus types. Second, hantavirus transmission is a complex ecological process, and residual confounding from unmeasured environmental and behavioral factors is possible. Third, underreporting is inevitable, as the data were obtained through passive surveillance. Additionally, changes over time in case detection, healthcare access, and public awareness may have led to differences in detection and reporting across periods, regions, or population subgroups in our long-term study, which may introduce bias and impact the completeness of the dataset. Despite these limitations, by incorporating a wide range of environmental and socioeconomic variables and analyzing long-term data, our study provides more nuanced insights into the determinants of HFRS transmission and the heterogeneity of risk patterns.

## Conclusions

In summary, this study systematically investigated the spatiotemporal dynamics of HFRS in Shandong province from 2005 to 2019, quantified the lagged and nonlinear associations between hydrometeorological conditions and HFRS, and assessed the modifying effects of regional characteristics. Our findings indicate that both extreme wet and dry conditions increase HFRS risk, with different lag periods. Disparities in county-level characteristics further amplify the adverse effects of hydrometeorological conditions on HFRS. These results provide an improved framework for understanding HFRS risk from a spatiotemporal perspective and offer useful guidance for public health policy and practice.

Based on these findings, we recommend that public health authorities strengthen HFRS surveillance and early warning systems during periods of extreme wet or dry conditions, with particular attention to lag periods identified. Targeted prevention and control measures, such as intensified rodent management, health education, and timely resource allocation, should be prioritized in economically disadvantaged and other high risks regions. Additionally, integrating hydrometeorological indicators into the existing public health monitoring and response systems will help support adaptive and proactive control strategies for HFRS control in the context of climate change.

## Supporting information

S1 Text Supplementary material and methods. (DOCX)

S1 Table Data on environmental factors affecting HFRS distribution. The base map is from the data center for geographic sciences and natural sources research, CAS (http://www.resdc.cn/data.aspx?DATAID=201).
(DOCX)

S2 Table Description of six variables in Shandong Province, 2005–2019. (DOCX)

S3 Table Detailed model specifications for the spatiotemporal Bayesian models. (DOCX)

S4 Table Maximum and cumulative risk of HFRS for extreme wet and extreme dry conditions within 6 months under high- and low-levels of county characteristics. (DOCX)

S1 Fig Spatial distribution of annual mean value of 136 county characteristics of Shandong provinces in China, 2005–2019. (TIF)

S2 Fig Spearman correlation coefficients of 136 county characteristics in Shandong provinces in China, 2005–2019. (TIF)

S3 Fig Spatial and temporal variation of HFRS incidence in Shandong by city. Note: Monthly HFRS incidence rate (per 100,000 people) between January, 2005, and December, 2019, aggregated at the city level (on a log + 1 transformed).
(TIF)

S4 Fig Spatial and temporal variation of monthly SPEI -1,3,6 at city level, 2005–2019. (TIF)

S5 Fig Spatial and temporal variation of monthly meteorological factors at city level, 2005–2019. (TIF)

S6 Fig Spatial Distribution of annual HFRS incidence in Shandong Province from 2005 to 2019. The base map is from the data center for geographic sciences and natural sources research, CAS (http://www.resdc.cn/data.aspx?DATAID=201).
(TIF)

S7 Fig Exposure-lag-response associations between climate indicators and HFRS. (A) Association between the risk of HFRS and temperature at different time lags. (B) Association between the risk of HFRS and relative humidity at different time lags. (C) Association between the risk of HFRS and precipitation at different time lags. The solid vertical line indicates the central values of climatic factors, and the dashed vertical lines represent the 2.5th and 97.5th percentiles of climatic factors.
(TIF)

S8 Fig Association between risk of HFRS and SPEI by gender, age and occupation. (TIF)

S9 Fig Sensitivity analysis results of exposure-lag risk of SPEI-6 on HFRS. (A) Main model; (B) climate factor was temperature; (C) climate factor was precipitation; (D) changing the *df* of exposure dimension in the cross basis of SPEI-6 to two; (E) changing the *df* of exposure dimension in the cross basis of SPEI-6 to four; (F) changing the *df* of lag dimension in the cross basis of SPEI-6 to four; (G) changing the *df* of lag dimension in the cross basis of SPEI-6 to five. (H) data in autumn-winter season; (I) data in spring season.
(TIF)

S10 Fig Observed versus posterior predictive HFRS incidence per city. Note: mean observed HFRS incidence (green curve) and corresponding posterior predictive mean HFRS incidence (solid pink curve) from January 2005 to December 2019 was estimated by Bayesian spatiotemporal models (refitted 12 x 15 times, leaving out one month per year at a time).
(TIF)

S11 Fig Association between risk of HFRS and SPEI at different time lags overall, and under high-, middle-, and low-county characteristics. (TIF)
