# Supplementary material for: Association between hydrometeorological conditions and hemorrhagic fever with renal syndrome in Shandong Province, China, from 2005 to 2019
Source: PLoS Negl Trop Dis. 2025 Jul 24;19(7):e0013306. doi: 10.1371/journal.pntd.0013306 (PMC12289069; doi:10.1371/journal.pntd.0013306)
Supplement: S1 Text — (DOCX) [file pntd.0013306.s001.docx]

**S1 Text. Supplementary material and methods**

**Computation of standardized precipitation evapotranspiration index**

Currently, several indices are used to monitor hydrological conditions such as droughts and floods, among which the Standardized Precipitation Index (SPI), the Standardized Precipitation Evapotranspiration Index (SPEI), and the Palmer Drought Severity Index (PDSI) are the most widely applied [1, 2]. SPI relies solely on precipitation and does not account for temperature, evapotranspiration, or soil moisture, which can limit its accuracy. PDSI incorporates evapotranspiration but depends on a soil moisture balance model. In comparison, SPEI considers both precipitation and potential evapotranspiration, providing a more comprehensive measure of hydrometeorological conditions and can be calculated for different time scales [3].

The SPEI combines the statistical approach of the SPI and the water balance concept of the PDSI, enabling it to capture variations in both precipitation and evapotranspiration. The calculation of the SPEI starts with the estimation of potential evapotranspiration (PET) based on the Thornthwaite method [4], which uses mean temperature and latitude as inputs. The formula is as follows:

$\begin{aligned} {PET}_{i}=16\times\left( \frac{10T_{i}}{H} \right)^{A} \end{aligned}$

$$\begin{aligned} H_{i}=\left( \frac{T_{i}}{5} \right)^{1.514} \end{aligned}$$

$$\begin{aligned} H=\sum_{i=1}^{12} H_{i} \end{aligned}$$

$$\begin{aligned} A=6.75\times{10}^{-7}H^{3}-7.71\times{10}^{-5}H^{2}+1.792\times{10}^{-2}H+0.49 \end{aligned}$$

where ${PET}_{i}$ represents monthly potential evapotranspiration (mm) for month *i*, $T_{i}$ represents monthly average temperature (℃), *A* is a constant, $H_{i}$ is the monthly heat index and $H$ is the annual heat index. When $T_{i}$≤ 0, $H_{i}$= 0, ${PET}_{i}$is set to 0 mm.

Next, the difference ($D_{i}$) between precipitation and PET is calculated as follows:

$$\begin{aligned} D_{i}=P_{i}-{PET}_{i} \end{aligned}$$

Subsequently, $D_{i}$is standardized to calculate the SPEI for each month. A log-logistic probability distribution with three parameters is used to address the possible negative values in $D_{i}$ series. The probability distribution function is expressed as:

$$\begin{aligned} F\left( x \right)=\left[ 1+\left( \frac{\alpha}{\gamma} \right)^{\beta} \right]^{-1} \end{aligned}$$

where the parameters $\alpha$, $\beta$ and $\gamma$ are estimated using the L-moments method:

$$\begin{aligned} \alpha=\frac{\left（ W_{0}-2W_{1} \right）}{\Gamma(1+1/{\beta)\Gamma(1-1/{\beta)}}} \end{aligned}$$

$$\begin{aligned} \beta=\frac{\left( 2W_{1}-W_{0} \right)}{\left( 6W_{1}-W_{0}-6W_{2} \right)} \end{aligned}$$

$$\begin{aligned} \gamma=W_{0}-\alpha\Gamma(1+1/{\beta)\Gamma(1-1/{\beta)}} \end{aligned}$$

$$\begin{aligned} W_{s}=\frac{1}{N}\sum_{i=1}^{N} \left( 1-F_{i} \right)^{s}D_{i} \end{aligned}$$

$$\begin{aligned} F_{i}=\frac{i-0.35}{N} \end{aligned}$$

where $\Gamma$ denotes the gamma function, $W_{0}, W_{1}$ and $W_{2}$ were the probability-weighted moments of the original $D_{i}$series, *N* is the number of months and $F_{i}$is the estimated frequency.

The cumulative probability is then standardized as follows:

$$\begin{aligned} P=1-F\left( x \right) \end{aligned}$$

Finally, the SPEI value were calculated:

$$\begin{aligned} SPEI=\left\{ \begin{aligned} W-\frac{c_{0}-c_{1}W+c_{2}W^{2}}{1+d_{1}W+d_{2}W^{2}+d_{3}W^{3}} P\leq0.5 \\ -(W-\frac{c_{0}-c_{1}W+c_{2}W^{2}}{1+d_{1}W+d_{2}W^{2}+d_{3}W^{3}}) P>0.5 \end{aligned} \right. \end{aligned}$$

$$\begin{aligned} W=\sqrt{-2\ln\left( P \right)} \end{aligned}$$

where the constants values are as follows: $c_{0}$= 2.515517, $c_{1}$ = 0.802853, $d_{1}$= 1.432788, $d_{2}$= 0.189269, $d_{3}$ = 0.001308.

**Spatiotemporal Bayesian hierarchical model**

The spatiotemporal Bayesian hierarchical model is a statistical framework that integrates Bayesian inference with a hierarchical structure, and is suitable for analyzing complex data with spatiotemporal dependencies [5]. This model incorporated spatiotemporal random effects to capture unobserved and unmeasured sources variation characteristics of data, as well as spatial and temporal dependence structures. Specifically, the model utilizes a hierarchical structure to combine the disease data, latent spatiotemporal effects, and prior information, thereby enabling comprehensive modeling of spatiotemporal associations.

In this study, a spatiotemporal Bayesian hierarchical model was constructed to examine the association between hydrometeorological conditions and HFRS in 136 counties from January 2005 to December 2019. A negative binomial distribution was applied to address the apparent overdispersion in HFRS cases counts [6]. The model was specified as follows:

$$\begin{aligned} Y_{it}\sim NegBin\left( \mu_{it},\kappa\right) \end{aligned}$$

$$\begin{aligned} \log{(\mu}_{it})=\log{(pop}_{ia(t)})+\log{(p}_{it}) \end{aligned}$$

where$Y_{it}$ is the count of HFRS cases in country i (i = 1, 2, …,136) during month *t* (*t*=1, 2, …, 180), $\mu_{it}$ is the corresponding mean of distribution, which is equal to the annual population per 100,000 ${pop}_{ia\left( t \right)} (a\left( t \right)=2005, 2006, \ldots, 2019)$ multiplied by the unknown HFRS incidence rate $p_{it}$ for county *i* at month *t* [7]. κ represents the scale or overdispersion parameter. Population effects were modelled by including the log of the population as an offset term in the linear predictor scale. We first constructed a baseline model incorporating spatiotemporal random effects to account for seasonality and interannual variability in spatial dependency:

$$\begin{aligned} \log\left( p_{it} \right)=\alpha+\beta_{s\left( i \right)m\left( t \right)}+\upsilon_{ia(t)}+\varphi_{ia(t)}+\delta_{it} \end{aligned}$$

where α is the intercept, $\beta_{s\left( i \right)m\left( t \right)}$ represents the city-level monthly random effect of month $m\left( t \right)$ for county *i* within city *s*. When incorporating monthly random effects, it is important to recognize that including these effects at the county level may lead to model overfitting. Here, *m(t)* = 1, …, 12 (corresponding to January through December) is used to account for the seasonality and seasonal autocorrelation. The monthly random effect employs a cyclic first-order random walk prior, enabling monthly HFRS incidence rate to depend on previous month, and linking January of year *a (t)* to December of year *a (t-1)* to maintain periodicity and capture seasonal cycles [7]. $\upsilon_{ia(t)}\mathrm{and}\varphi_{ia(t)}$ represent the structured and unstructured spatial random effects at county-level, respectively. Structured spatial effect is included to capture spatial correlations among neighboring regions due to shared or correlated environmental and socioeconomic factors (such as climate zones, land use patterns, etc.). In contrast, the unstructured spatial effect captures spatial heterogeneity driven by unique, non-spatial characteristics of individual units, accounting for unknown or unmeasured confounders, such as differences in healthcare access, vector ecology, and reporting rates. The prior distributions for spatial effects are specified using a modified Besag-York-Mollié (BYM) model. The modified BYM model introduces two key parameters: the precision parameter, which controls the overall marginal variance explained by the spatial effects, and the mixing parameter, which allocates the existing variance between the structured and unstructured spatial components [8]. For all random effects, penalized complexity (PC) priors were adopted. The precision parameter was defined as $\tau=1/\sigma^{2}$, with *Pr* ($1/\surd\tau>0.5$) = 0.01. $\delta_{it}$ is a space-time interaction term to captuzre residual spatiotemporal variation, representing temporal variation in the spatial effect of disease incidence. This term is assumed to follow a normal prior distribution: $\delta_{it}\sim N(0,\sigma_{\delta}^{2})$.

**Distributed Lag Non-linear Model**

To quantify the association between SPEI and HFRS incidence, the model further incorporated a Distributed Lag Non-linear Model (DLNM). The DLNM based on the framework of generalized linear models and generalized additive models, employs cross-basis functions to evaluate the exposure–response and lag–response between variables [9]. This model can fit both the nonlinear exposure–response relationships and the lagged effects within the exposure–outcome association, and has been widely applied in environmental health research.

In this study, a stepwise strategy was employed to estimate the association between HFRS and climate indicators, progressively assessing the modification role of county-specific characteristics in this association. Firstly, the cross-basis functions of three SPEI variables (SPEI-1, SPEI-3, and SPEI-6) were individually introduced in the baseline model to construct the baseline-SPEI model:

$$\begin{aligned} \log\left( p_{it} \right)=\alpha+\beta_{s\left( i \right)m\left( t \right)}+\upsilon_{ia(t)}+\varphi_{ia(t)}+\delta_{it}+cb\left( SPEI,l \right) \end{aligned}$$

where *SPEI* represents one of the three selected SPEI variables, and $cb\left( SPEI,l \right)$ denotes the cross-basis function for the corresponding SPEI indicator. After model construction, model fit was evaluated using the Deviance Information Criterion (DIC), and the model with the lowest DIC value was selected as the optimal hydrometeorological model [10].

Subsequently, based on the optimal hydrometeorological model, cross-basis functions for three meteorological variables (mean temperature, relative humidity, and cumulative precipitation) were incorporated individually to construct the combined meteorological and hydrometeorological model:

$$\begin{aligned} \log\left( p_{it} \right)=\alpha+\beta_{s\left( i \right)m\left( t \right)}+\upsilon_{ia(t)}+\varphi_{ia(t)}+\delta_{it}+cb\left( SPEI,l \right)+cb\left( met,l \right) \end{aligned}$$

where *met* represents one of the three meteorological factors, and $cb(met,l)$ represents the cross-basis function of each meteorological factor. The model with the lowest DIC value was selected as the best-fitting model. Considering the incubation period of HFRS and the potential delays in pathogen transmission among rodents and external environment, the lag period was set from 0-6 months to explore all possible lagged association [11, 12]. To accurately capture potentially complex associations between climatic indicators and their lagged effects on HFRS risk, natural cubic splines with 3 degrees of freedom were applied to both the exposure and lag dimensions. Model parameters were estimated within a Bayesian framework using the Integrated Nested Laplace Approximation (INLA) method [13].

To investigate the potential modification effect of county-level characteristics on the SPEI–HFRS incidence association, this study selected six indicators based on the biological characteristics of HFRS and data availability: population density, per capita Gross Domestic Product (GDP), Normalized Difference Vegetation Index (NDVI), elevation and Total Power of Agricultural Machinery (TPAM). We introduced a linear interaction term between $cb\left( SPEI,l \right)$ and each county indicator into the final model. Each indicator was centered at the 10^th^, 50^th^ and 90^th^ percentiles of the 136 counties to assess the effect of SPEI-6 at different levels of county characteristics. Within the interaction models, the exposure-lag-response association varies according to the value of the modifying variable. Adjusting the centering value does not alter the model structure but results in an alternative parameterization [7].

To evaluate the performance of the model, we generated posterior predictive distributions for the response variable by utilizing samples from the posterior distributions of both the model parameters and hyperparameters. The model was fitted 15 × 12 times, with each iteration excluding a month per year. For each prediction month, we estimated the posterior predictive distribution of HFRS incidence by sampling 1,000 values from a negative binomial distribution, where the mean was based on the fitted model’s predictions and the scale parameter was taken from the estimated overdispersion. This approach incorporates parameter uncertainty into the predicted outcomes. Posterior predictive distributions were produced for each time period and county, and the resulting predictive summaries were compared to the actual observed case counts.

**References**

1. Mckee TB, Doesken NJ, Kleist J. The relationship of drought frequency and duration of time scales. Eight Conference on Apllied Climatology, American Meteorological Society, Jan 17–23. 1993.

2. Vicente-Serrano SM, Beguería S, López-Moreno JI. A Multiscalar Drought Index Sensitive to Global Warming: The Standardized Precipitation Evapotranspiration Index. Journal of Climate. 2010;23(7):1696-718. doi: https: 10.1175/2009JCLI2909.1.

3. 中华人民共和国国家质量监督检验检疫总局. 气象干旱等级(GB/T20481-2017). 2017.

4. Thornthwaite CW. An Approach Toward a Rational Classification of Climate. Soil Science. 1948;66:55-94.

5. 李世元, 王学梅. 贝叶斯时空模型在空间流行病学中的研究进展. 世界最新医学信息文摘. 2017;17(34):55-7.

6. Li G, Haining R, Richardson S, Best N. Space–time variability in burglary risk: A Bayesian spatio-temporal modelling approach. Spatial Statistics. 2014;9:180-91. doi: https://doi.org/10.1016/j.spasta.2014.03.006.

7. Lowe R, Lee SA, O'Reilly KM, Brady OJ, Bastos L, Carrasco-Escobar G, et al. Combined effects of hydrometeorological hazards and urbanisation on dengue risk in Brazil: a spatiotemporal modelling study. The Lancet Planetary Health. 2021;5(4):e209-e19. doi: https://doi.org/10.1016/S2542-5196(20)30292-8.

8. Riebler A, Sørbye SH, Simpson D, Rue H. An intuitive Bayesian spatial model for disease mapping that accounts for scaling. Stat Methods Med Res. 2016;25(4):1145-65. doi: 10.1177/0962280216660421.

9. Gasparrini A, Armstrong B, Kenward MG. Distributed lag non-linear models. Stat Med. 2010;29(21):2224-34. doi: 10.1002/sim.3940.

10. Spiegelhalter DJ, Best NG, Carlin BP, Van Der Linde A. Bayesian Measures of Model Complexity and Fit. Journal of the Royal Statistical Society Series B: Statistical Methodology. 2002;64(4):583-639. doi: 10.1111/1467-9868.00353.

11. Cao L, Huo X, Xiang J, Lu L, Liu X, Song X, et al. Interactions and marginal effects of meteorological factors on haemorrhagic fever with renal syndrome in different climate zones: Evidence from 254 cities of China. Sci Total Environ. 2020;721:137564. Epub 20200225. doi: 10.1016/j.scitotenv.2020.137564.

12. Lv CL, Tian Y, Qiu Y, Xu Q, Chen JJ, Jiang BG, et al. Dual seasonal pattern for hemorrhagic fever with renal syndrome and its potential determinants in China. Sci Total Environ. 2023;859(Pt 2):160339. Epub 20221122. doi: 10.1016/j.scitotenv.2022.160339.

13. Rue H, Martino S, Chopin N. Approximate Bayesian inference for latent Gaussian models by using integrated nested Laplace approximations. Journal of the Royal Statistical Society: Series B (Statistical Methodology). 2009;71(2):319-92. doi: 10.1111/j.1467-9868.2008.00700.x.
