# Supplementary material for: Association between hydrometeorological conditions and hemorrhagic fever with renal syndrome in Shandong Province, China, from 2005 to 2019
Source: PLoS Negl Trop Dis. 2025 Jul 24;19(7):e0013306. doi: 10.1371/journal.pntd.0013306 (PMC12289069; doi:10.1371/journal.pntd.0013306)
Supplement: S1 Table — The base map is from the data center for geographic sciences and natural sources research, CAS (http://www.resdc.cn/data.aspx?DATAID=201). (DOCX) [file pntd.0013306.s002.docx]

**S1 Table.** Data on county characteristics affecting HFRS distribution.

| **Category** | **Variable** |
| --- | --- |
| Economic development | Nighttime-light |
|  | Population density |
|  | Per capita GDP |
| Climate factors | Annual Temperature |
|  | Annual Humidity |
|  | Annual Precipitation |
| Environmental factors | NDVI |
|  | Cropland area |
|  | Forest area |
|  | Grassland area |
|  | Water area |
|  | Impervious area |
| Geographical factors | Elevation |
| Agricultural environment | TPAM |

TPAM: total power of agricultural machinery.
