# Supplementary material for: Association between hydrometeorological conditions and hemorrhagic fever with renal syndrome in Shandong Province, China, from 2005 to 2019
Source: PLoS Negl Trop Dis. 2025 Jul 24;19(7):e0013306. doi: 10.1371/journal.pntd.0013306 (PMC12289069; doi:10.1371/journal.pntd.0013306)
Supplement: S2 Table — (DOCX) [file pntd.0013306.s003.docx]

**S2 Table.** Description of six variables in Shandong Province, 2005-2019.

| Variable | Minimum | Median | IQR | Maximum |
| --- | --- | --- | --- | --- |
| Population density | 117.68 | 608.49 | 303.13 | 22780.19 |
| Per capita GDP | 0.39 | 3.83 | 4.00 | 23.62 |
| Temperature | 11.04 | 13.97 | 1.46 | 16.36 |
| NDVI | 0.25 | 0.73 | 0.10 | 0.85 |
| Elevation | 2.59 | 52.5 | 70.64 | 435.41 |
| TPAM | 0.00 | 77.22 | 69.07 | 336.00 |

IQR: Inter-quartile range.
