# Supplementary material for: Association between hydrometeorological conditions and hemorrhagic fever with renal syndrome in Shandong Province, China, from 2005 to 2019
Source: PLoS Negl Trop Dis. 2025 Jul 24;19(7):e0013306. doi: 10.1371/journal.pntd.0013306 (PMC12289069; doi:10.1371/journal.pntd.0013306)
Supplement: S3 Table — (DOCX) [file pntd.0013306.s004.docx]

**S3 Table.** Detailed model specifications for the spatiotemporal Bayesian models.

| Model | Equation | DIC | CV log score |
| --- | --- | --- | --- |
| Baseline model | $\log\left( \mu_{it} \right)=\alpha+\beta_{s\left( i \right)m\left( t \right)}+\upsilon_{ia(t)}+\varphi_{ia(t)}+\delta_{it}$ | 43907 | 0.900 |
| Hydrological model | | | |
| Baseline model + SPEI-1 | $\log\left( \mu_{it} \right)=\alpha+\beta_{s\left( i \right)m\left( t \right)}+\upsilon_{ia(t)}+\varphi_{ia(t)}+\delta_{it}+cb(SPEI-1,l)$ | 43770 | 0.896 |
| Baseline model + SPEI-3 | $\log\left( \mu_{it} \right)=\alpha+\beta_{s\left( i \right)m\left( t \right)}+\upsilon_{ia(t)}+\varphi_{ia(t)}+\delta_{it}+cb(SPEI-3,l)$ | 43796 | 0.897 |
| Baseline model + SPEI-6 | $\log\left( \mu_{it} \right)=\alpha+\beta_{s\left( i \right)m\left( t \right)}+\upsilon_{ia(t)}+\varphi_{ia(t)}+\delta_{it}+cb(SPEI-6,l)$ | 43729 | 0.895 |
| Hydrological and meteorological model | | | |
| Baseline model + SPEI-6 + Temp | $\log\left( \mu_{it} \right)=\alpha+\beta_{s\left( i \right)m\left( t \right)}+\upsilon_{ia(t)}+\varphi_{ia(t)}+\delta_{it}+cb(SPEI-6,l) +cb(Temp,l)$ | 43742 | 0.896 |
| Baseline model + SPEI-6 + Hum | $\log\left( \mu_{it} \right)=\alpha+\beta_{s\left( i \right)m\left( t \right)}+\upsilon_{ia(t)}+\varphi_{ia(t)}+\delta_{it}+cb(SPEI-6,l) +cb(Hum,l)$ | 43598 | 0.892 |
| Baseline model + SPEI-6 + Pre | $\log\left( \mu_{it} \right)=\alpha+\beta_{s\left( i \right)m\left( t \right)}+\upsilon_{ia(t)}+\varphi_{ia(t)}+\delta_{it}+cb(SPEI-6,l) +cb(Pre,l)$ | 43637 | 0.893 |

Note: The baseline model comprised an intercept, α, city-specific monthly random effects, $\beta_{i\left( i \right)m\left( t \right)}$, Specific spatially unstructured, $\nu_{i}$, and structured, $\upsilon_{i}$, random effects at the city level, a space-time interaction term, $\delta_{it}$. The final model additionally added the cross-basis functions of SPEI-6. DIC, deviance information criterion; CV, cross-validated; SPEI, standardized precipitation evapotranspiration index; cb, cross-basis. Temp, monthly mean temperature; Hum, monthly mean relative humidity; Pre, monthly cumulative precipitation.
