# Supplementary material for: Association between hydrometeorological conditions and hemorrhagic fever with renal syndrome in Shandong Province, China, from 2005 to 2019
Source: PLoS Negl Trop Dis. 2025 Jul 24;19(7):e0013306. doi: 10.1371/journal.pntd.0013306 (PMC12289069; doi:10.1371/journal.pntd.0013306)
Supplement: S4 Table — (DOCX) [file pntd.0013306.s005.docx]

**S4 Table.** Maximum and cumulative risk of HFRS for extreme wet and extreme dry conditions within 6 months under high- and low-levels of county characteristics.

|  | Wet conditions: | | | | | | Dry conditions: | | | | | |
| --- | --- | --- | --- | --- | --- | --- | --- | --- | --- | --- | --- | --- |
|  | SPEI-6 >0 | | | SPEI-6 =2 | | | SPEI-6 <0 | | | SPEI-6 =-2 | | |
|  | Maximum SPEI-6, RR and lag | | | Maximum RR and lag | | Cumulative RR (95% CI) | Maximum SPEI-6, RR and lag | | | Maximum RR and lag | | Cumulative RR (95% CI) |
|  | SPEI | RR (95% CI) | lag | RR (95% CI) | lag |  | SPEI | RR (95% CI) | lag | RR (95% CI) | lag |  |
| Overall | 2.4 | 1.69 (1.50,1.90) | 6 | 1.49 (1.37,1.63) | 6 | 1.82 (1.50,2.21) | -3.9 | 1.23 (0.89,1.70) | 6 | 1.05 (1.01,1.09) | 5 | 1.10 (0.91,1.33) |
| Population Density | | | | | | | | | | | | |
| High | 2.4 | 1.71 (1.48,1.97) | 6 | 1.50 (1.35,1.65) | 6 | 1.86 (1.48,2.35) | -3.9 | 1.11 (0.92,1.34) | 2 | 1.05 (1.01,1.09) | 4 | 1.08 (0.88,1.31) |
| Medium | 2.4 | 1.68 (1.49,1.89) | 6 | 1.49 (1.37,1.62) | 6 | 1.82 (1.50,2.21) | -3.9 | 1.28 (0.93,1.77) | 6 | 1.05 (0.97,1.14) | 6 | 1.08 (0.89,1.31) |
| Low | 2.4 | 1.67 (1.47,1.89) | 6 | 1.49 (1.36,1.62) | 6 | 1.81 (1.48,2.21) | -3.9 | 1.33 (0.96,1.86) | 6 | 1.06 (0.97,1.15) | 6 | 1.08 (0.89,1.31) |
| Per capita GDP | | | | | | | | | | | | |
| High | 2.4 | 1.25 (0.97,1.63) | 6 | 1.17 (0.97,1.41) | 6 | 1.49 (0.91,2.44) | -1.0 | 1.04 (1.01,1.07) | 4 | 0.99 (0.94,1.04) | 4 | 0.81 (0.63,1.05) |
| Medium | 2.4 | 1.62 (1.43,1.83) | 6 | 1.45 (1.33,1.58) | 6 | 1.72 (1.40,2.10) | -3.9 | 1.36 (0.96,1.93) | 6 | 1.07 (0.98,1.17) | 6 | 1.18 (0.96,1.45) |
| Low | 2.4 | 1.77 (1.51,2.06) | 6 | 1.56 (1.40,1.74) | 6 | 1.74 (1.39,2.19) | -3.9 | 1.70 (1.12,2.58) | 6 | 1.10 (0.99,1.23) | 6 | 1.35 (1.05,1.73) |
| Annual Temperature | | | | | | | | | | | | |
| High | 2.4 | 1.44 (1.13,1.83) | 6 | 1.32 (1.11,1.56) | 6 | 2.39 (1.65,3.48) | -1.4 | 1.04 (0.99,1.10) | 3 | 1.03 (0.96,1.11) | 3 | 0.91 (0.71,1.16) |
| Medium | 2.4 | 1.60 (1.40,1.83) | 6 | 1.43 (1.30,1.57) | 6 | 2.04 (1.65,2.53) | -1.6 | 1.03 (1.00,1.06) | 4 | 1.02 (0.98,1.07) | 4 | 0.90 (0.73,1.11) |
| Low | 2.4 | 1.79 (1.52,2.11) | 6 | 1.55 (1.38,1.75) | 6 | 1.66 (1.27,2.16) | -3.9 | 1.36 (0.88,2.09) | 6 | 1.09 (0.97,1.23) | 6 | 0.89 (0.71,1.12) |
| NDVI | | | | | | | | | | | | |
| High | 2.4 | 1.47 (1.28,1.70) | 6 | 1.35 (1.22,1.50) | 6 | 1.38 (1.09,1.76) | -3.9 | 1.13 (0.79,1.61) | 0 | 1.01 (0.96,1.06) | 4 | 0.87 (0.70,1.09) |
| Medium | 2.4 | 1.65 (1.46,1.86) | 6 | 1.47 (1.35,1.60) | 6 | 1.76 (1.44,2.14) | -3.9 | 1.14 (0.80,1.62) | 0 | 1.03 (0.99,1.07) | 4 | 1.00 (0.82,1.23) |
| Low | 2.4 | 2.15 (1.75,2.64) | 6 | 1.78 (1.53,2.06) | 6 | 3.12 (2.10,4.64) | -3.9 | 1.31 (0.94,1.83) | 6 | 1.10 (1.00,1.22) | 6 | 1.40 (1.11,1.77) |
| Elevation | | | | | | | | | | | | |
| High | 2.4 | 1.76 (1.48,2.09) | 6 | 1.57 (1.39,1.77) | 6 | 2.25 (1.69,2.98) | -3.9 | 1.87 (1.22,2.87) | 6 | 1.10 (0.98,1.22) | 6 | 1.34 (1.01,1.77) |
| Medium | 2.4 | 1.64 (1.43,1.89) | 6 | 1.45 (1.31,1.60) | 6 | 1.57 (1.25,1.97) | -3.9 | 1.13 (0.92,1.39) | 2 | 1.04 (1.00,1.09) | 4 | 1.08 (0.88,1.33) |
| Low | 2.4 | 1.61 (1.36,1.91) | 6 | 1.41 (1.25,1.60) | 6 | 1.44 (1.09,1.91) | -3.9 | 1.10 (0.86,1.41) | 2 | 1.04 (0.99,1.09) | 4 | 1.01 (0.80,1.29) |
| TPAM | | | | | | | | | | | | |
| High | 2.4 | 1.33 (1.10,1.59) | 6 | 1.22 (1.07,1.39) | 6 | 1.14 (0.84,1.54) | -0.8 | 1.03 (1.01,1.05) | 4 | 0.96 (0.88,1.05) | 6 | 0.64 (0.48,0.85) |
| Medium | 2.4 | 1.69 (1.50,1.91) | 6 | 1.50 (1.38,1.63) | 6 | 1.88 (1.55,2.28) | -3.9 | 1.16 (0.84,1.61) | 6 | 1.04 (1.00,1.09) | 4 | 1.01 (0.83,1.23) |
| Low | 2.4 | 2.07 (1.71,2.50) | 6 | 1.77 (1.54,2.04) | 6 | 2.88 (2.12,3.92) | -3.9 | 1.85 (1.19,2.88) | 6 | 1.12 (1.06,1.19) | 4 | 1.53 (1.19,1.99) |
