# Supplementary figures and images for: Association between hydrometeorological conditions and hemorrhagic fever with renal syndrome in Shandong Province, China, from 2005 to 2019
Source: PLoS Negl Trop Dis. 2025 Jul 24;19(7):e0013306. doi: 10.1371/journal.pntd.0013306 (PMC12289069; doi:10.1371/journal.pntd.0013306)

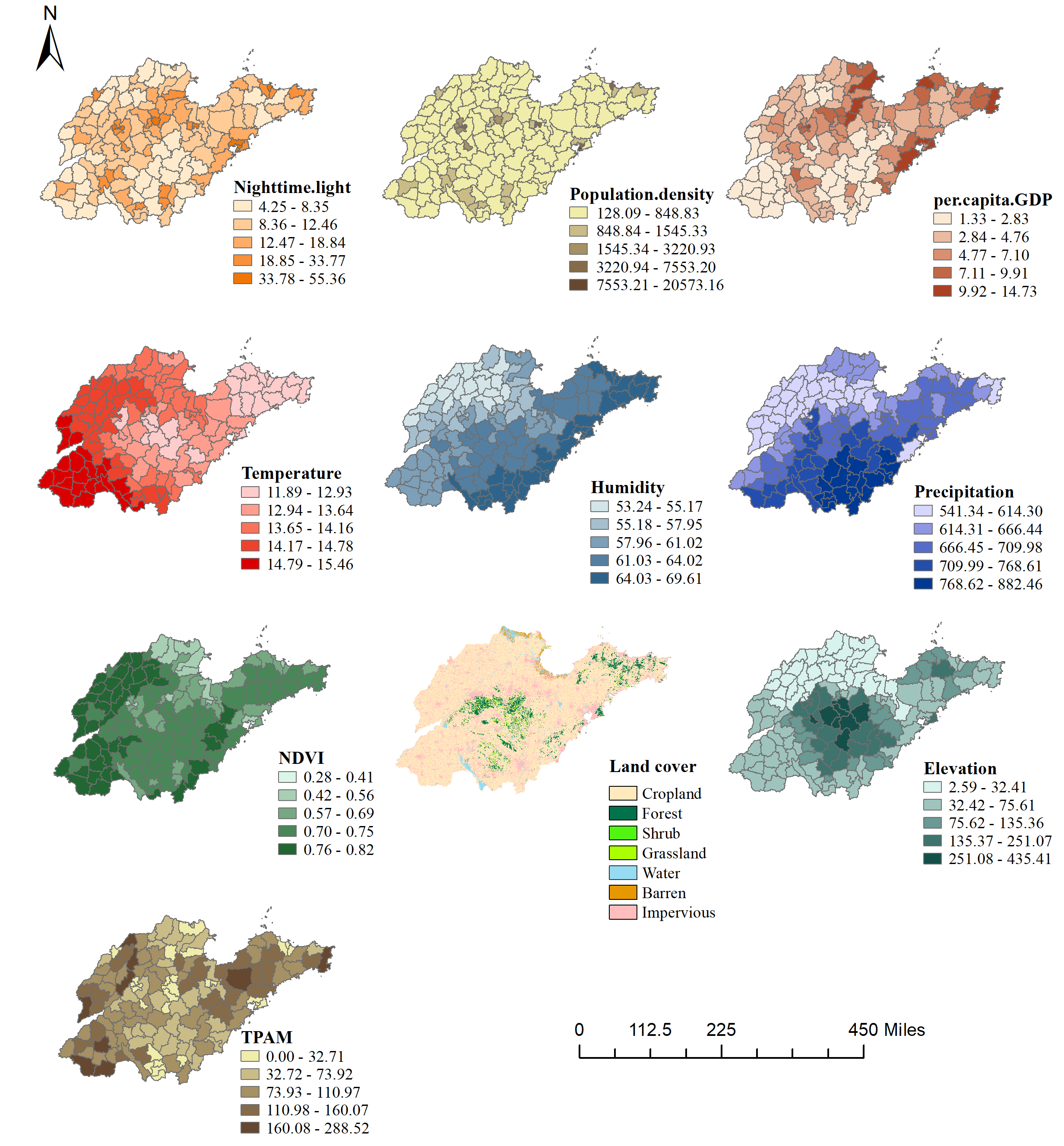

Supplement: S1 Fig — (TIF) [file pntd.0013306.s006.tif]

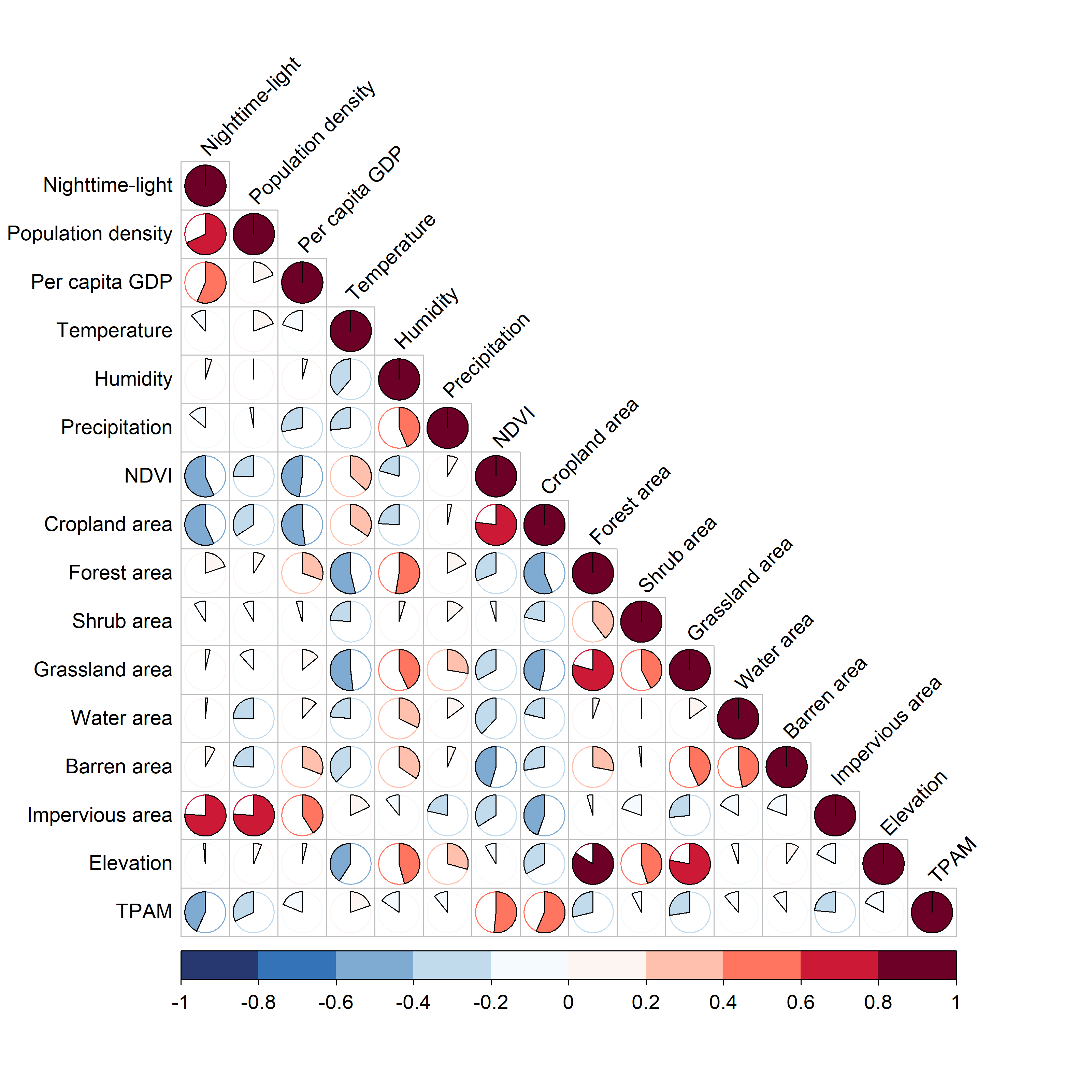

Supplement: S2 Fig — (TIF) [file pntd.0013306.s007.tif]

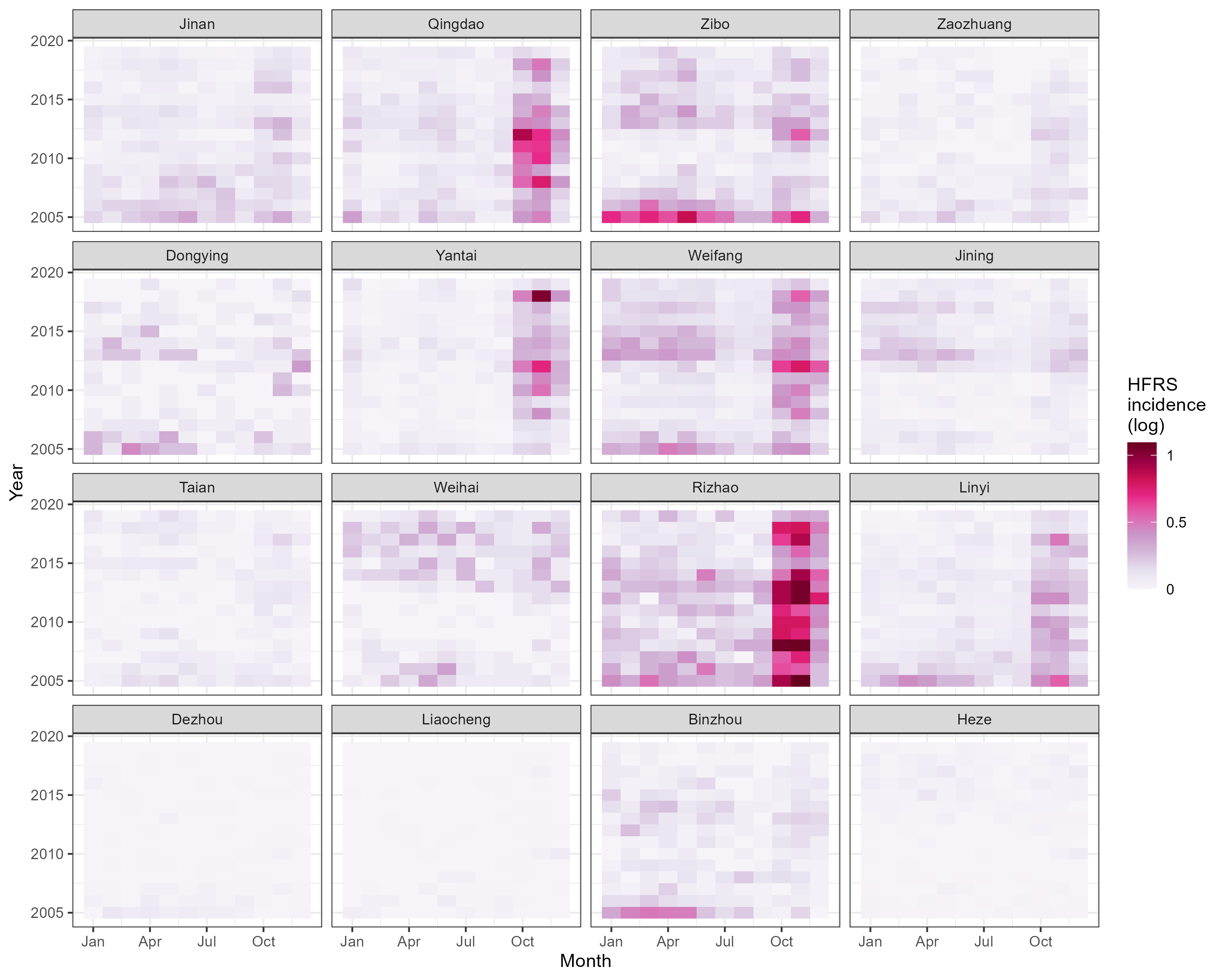

Supplement: S3 Fig — Note: Monthly HFRS incidence rate (per 100,000 people) between January, 2005, and December, 2019, aggregated at the city level (on a log + 1 transformed). (TIF) [file pntd.0013306.s008.tif]

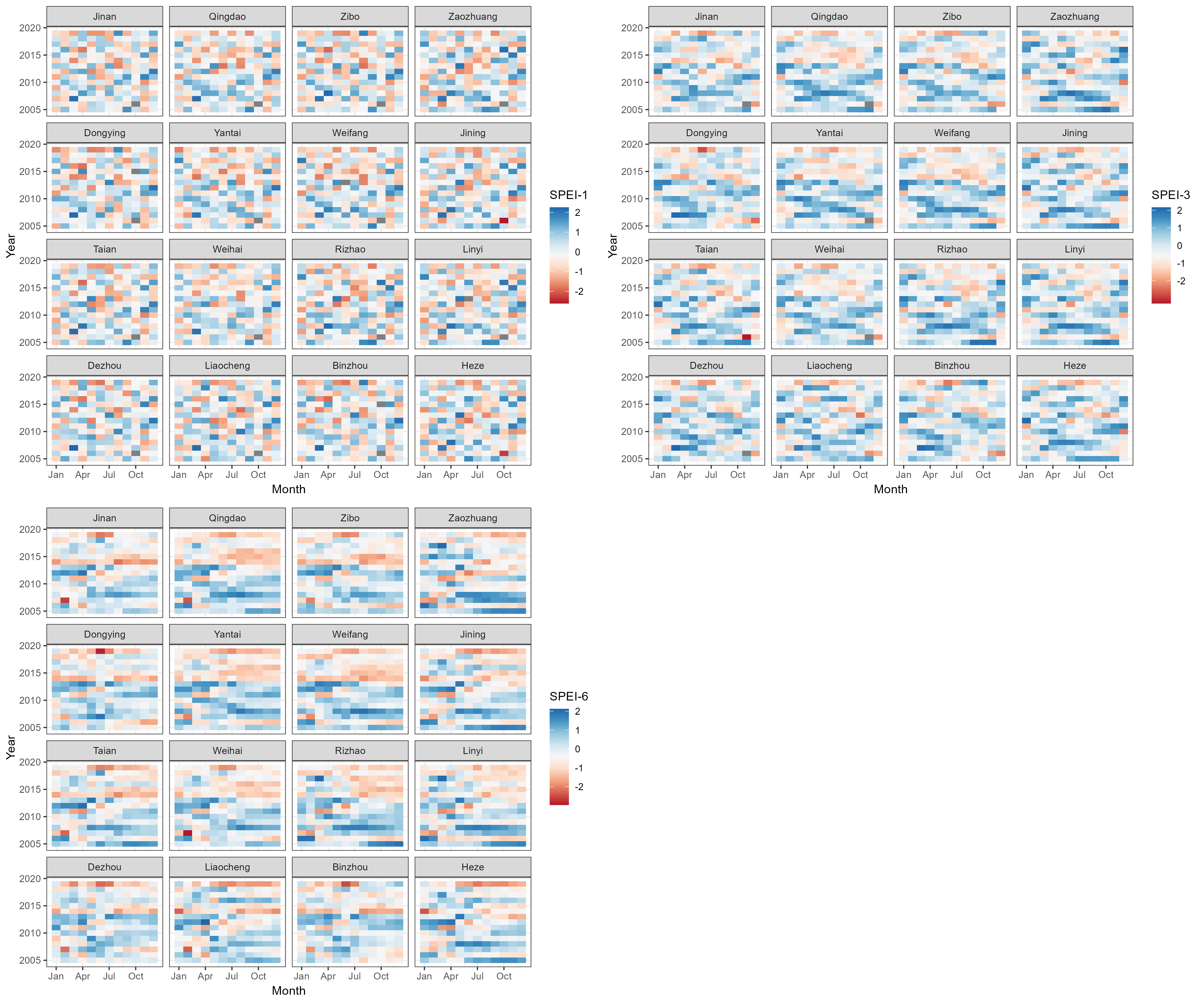

Supplement: S4 Fig — (TIF) [file pntd.0013306.s009.tif]

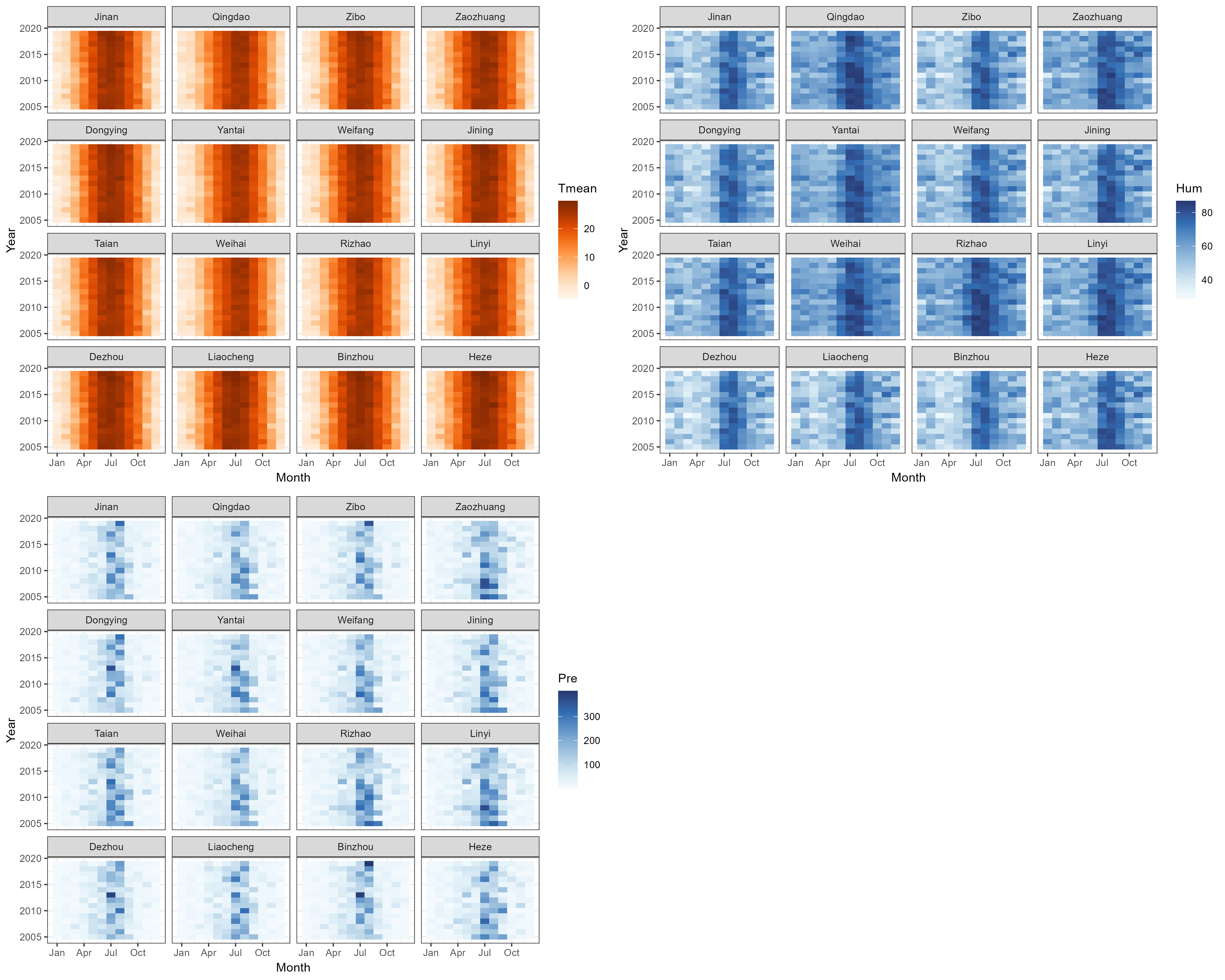

Supplement: S5 Fig — (TIF) [file pntd.0013306.s010.tif]

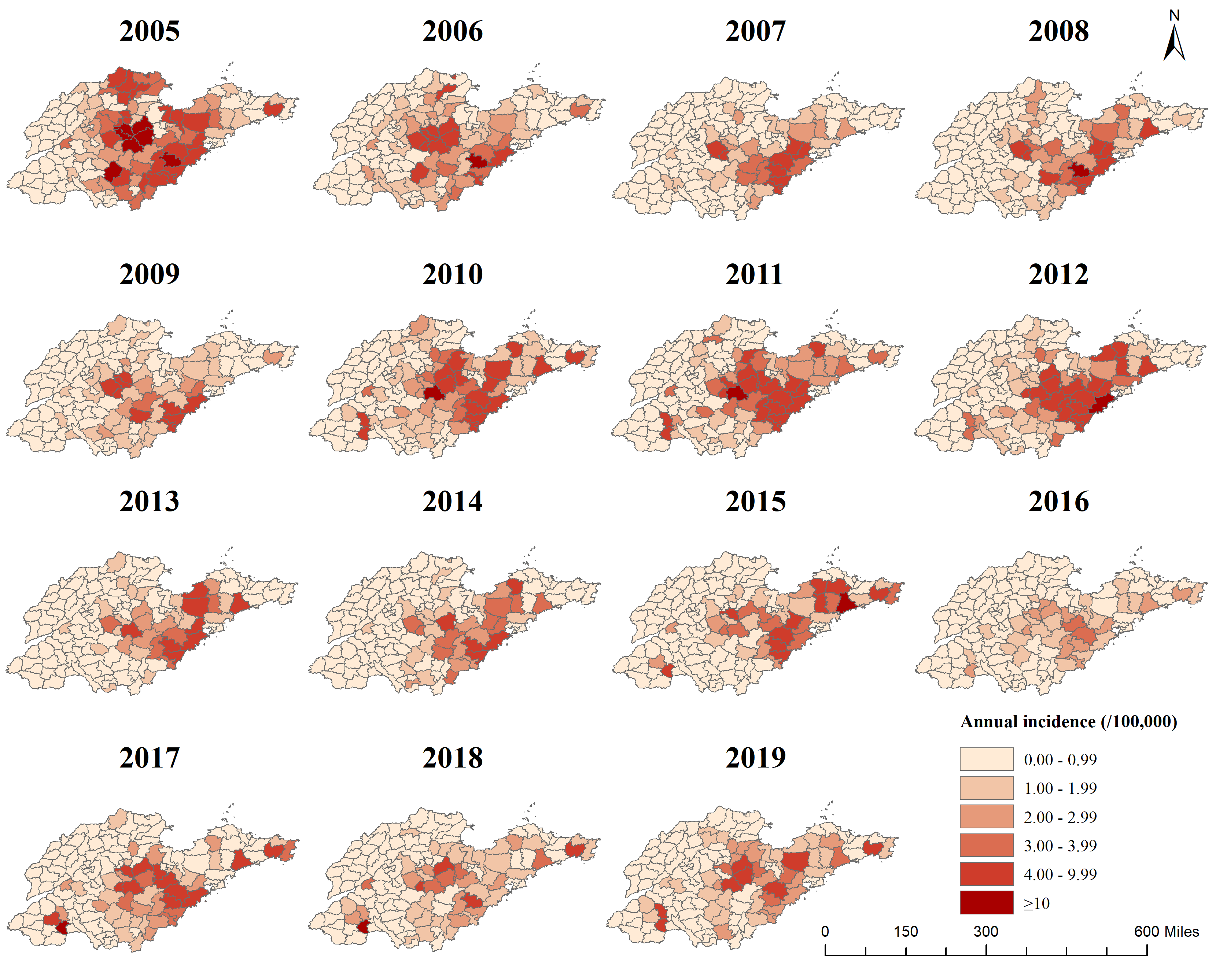

Supplement: S6 Fig — The base map is from the data center for geographic sciences and natural sources research, CAS (http://www.resdc.cn/data.aspx?DATAID=201). (TIF) [file pntd.0013306.s011.tif]

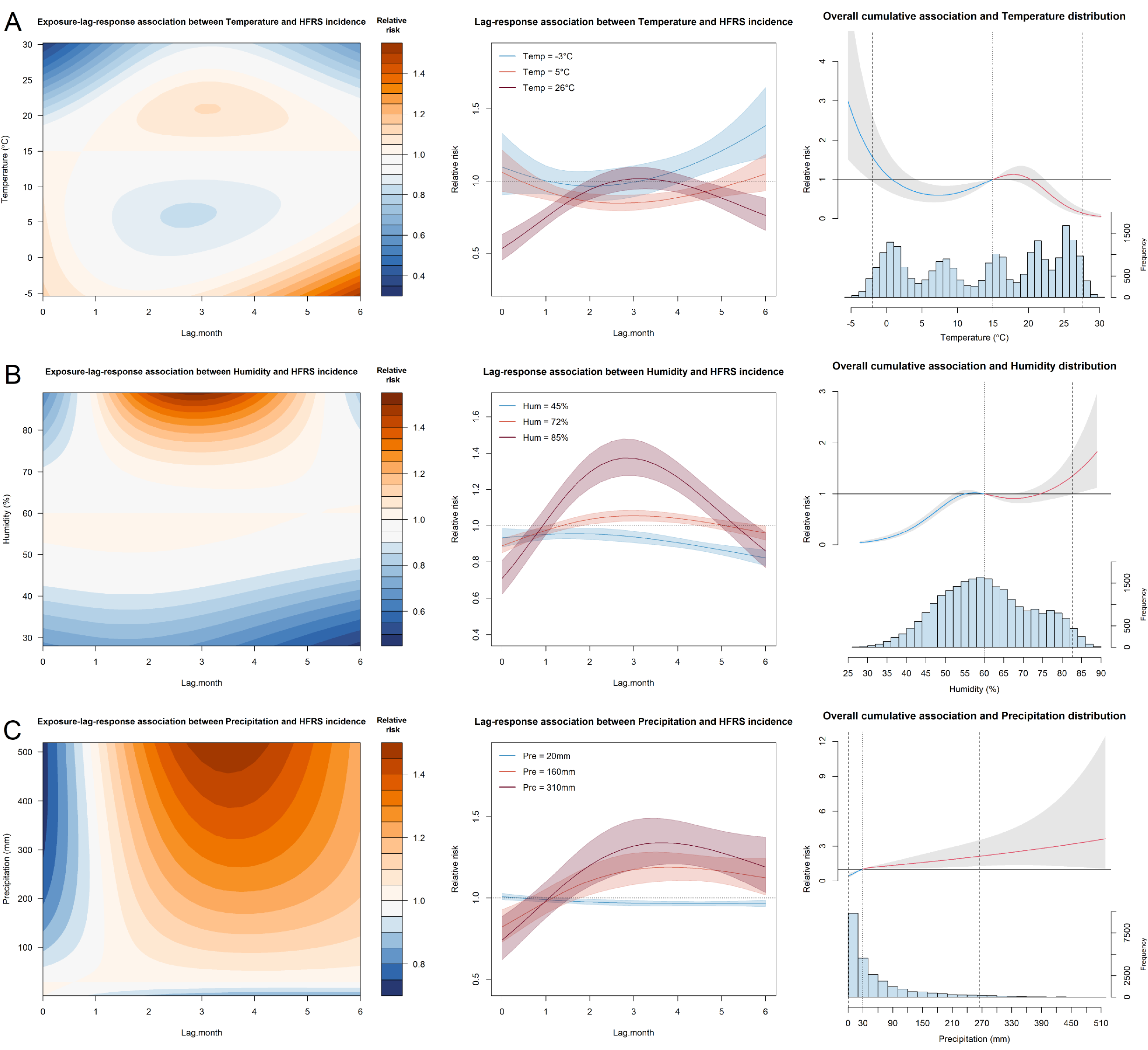

Supplement: S7 Fig — (A) Association between the risk of HFRS and temperature at different time lags. (B) Association between the risk of HFRS and relative humidity at different time lags. (C) Association between the risk of HFRS and precipitation at different time lags. The solid vertical line indicates the central values of climatic factors, and the dashed vertical lines represent the 2.5th and 97.5th percentiles of climatic factors. (TIF) [file pntd.0013306.s012.tif]

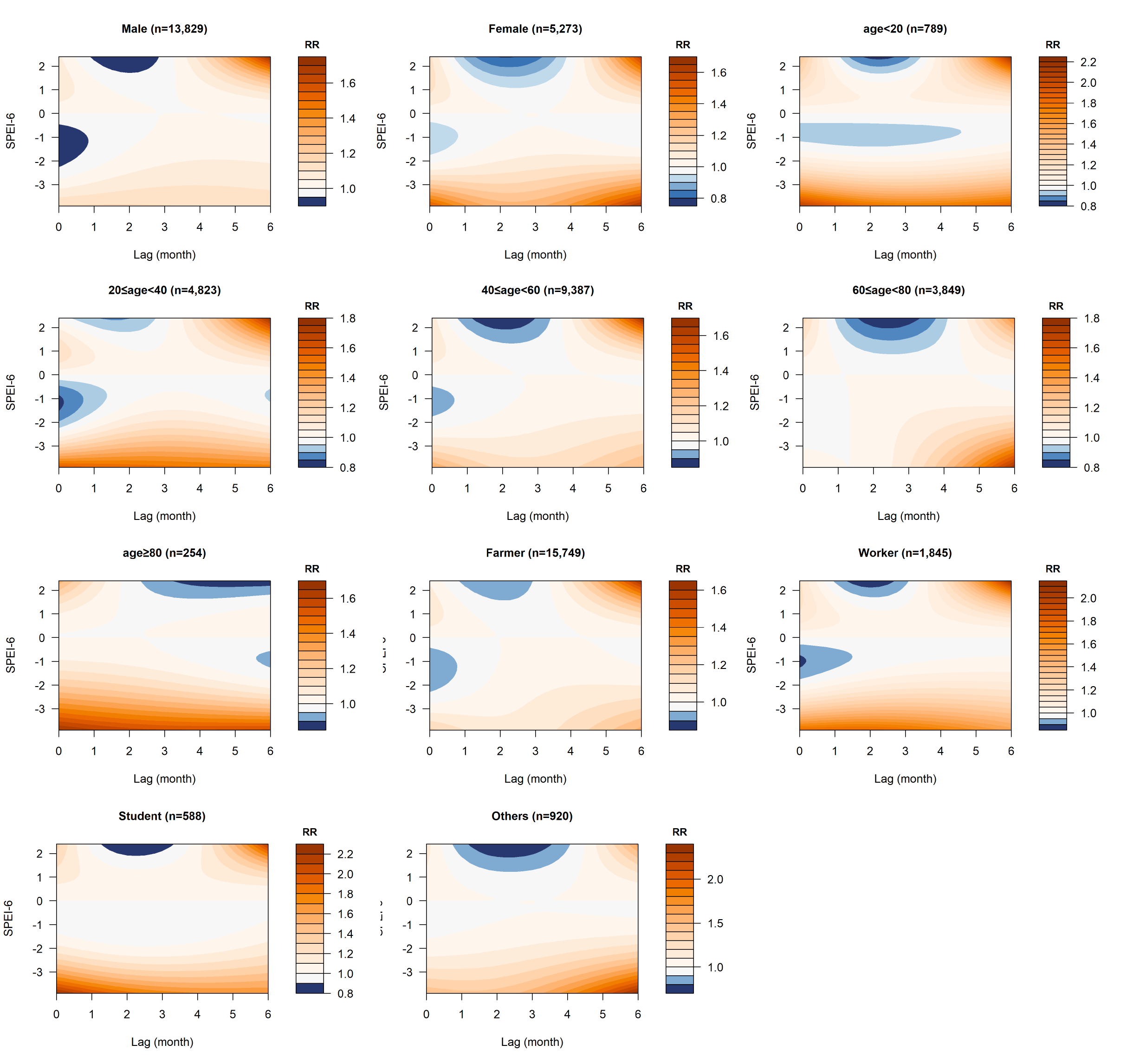

Supplement: S8 Fig — (TIF) [file pntd.0013306.s013.tif]

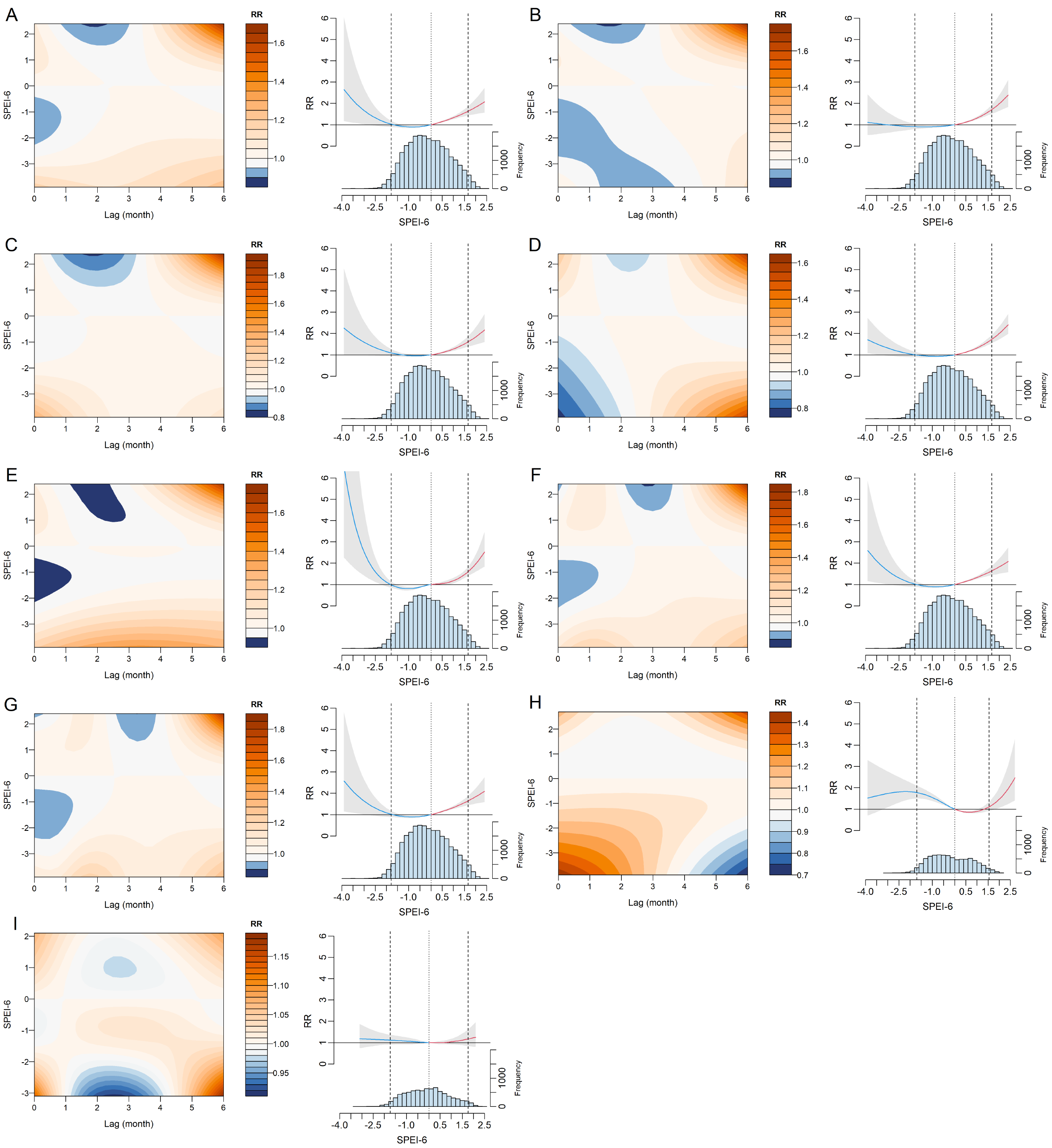

Supplement: S9 Fig — (A) Main model; (B) climate factor was temperature; (C) climate factor was precipitation; (D) changing the df of exposure dimension in the cross basis of SPEI-6 to two; (E) changing the df of exposure dimension in the cross basis of SPEI-6 to four; (F) changing the df of lag dimension in the cross basis of SPEI-6 to four; (G) changing the df of lag dimension in the cross basis of SPEI-6 to five. (H) data in autumn-winter season; (I) data in spring season. (TIF) [file pntd.0013306.s014.tif]

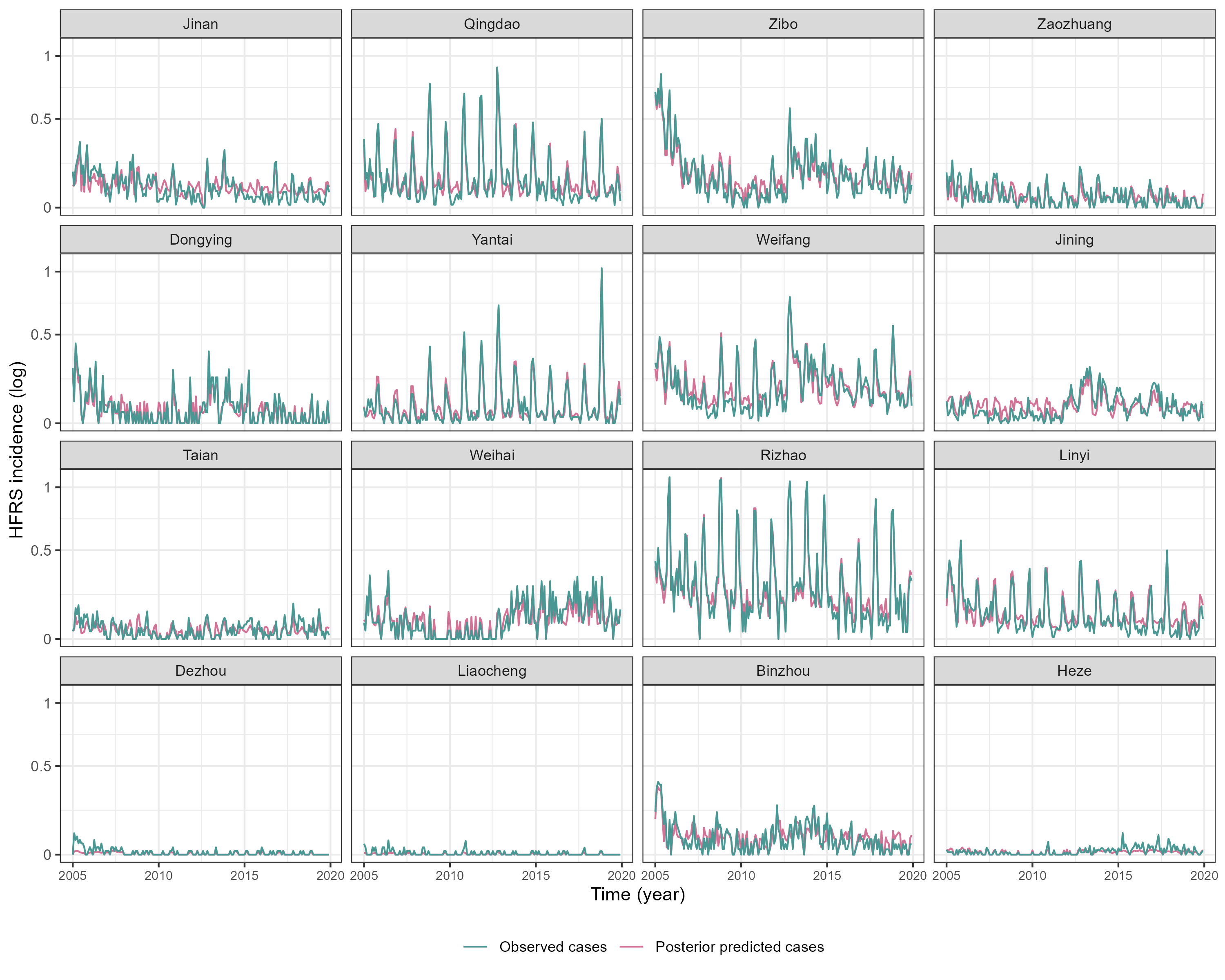

Supplement: S10 Fig — Note: mean observed HFRS incidence (green curve) and corresponding posterior predictive mean HFRS incidence (solid pink curve) from January 2005 to December 2019 was estimated by Bayesian spatiotemporal models (refitted 12 x 15 times, leaving out one month per year at a time). (TIF) [file pntd.0013306.s015.tif]

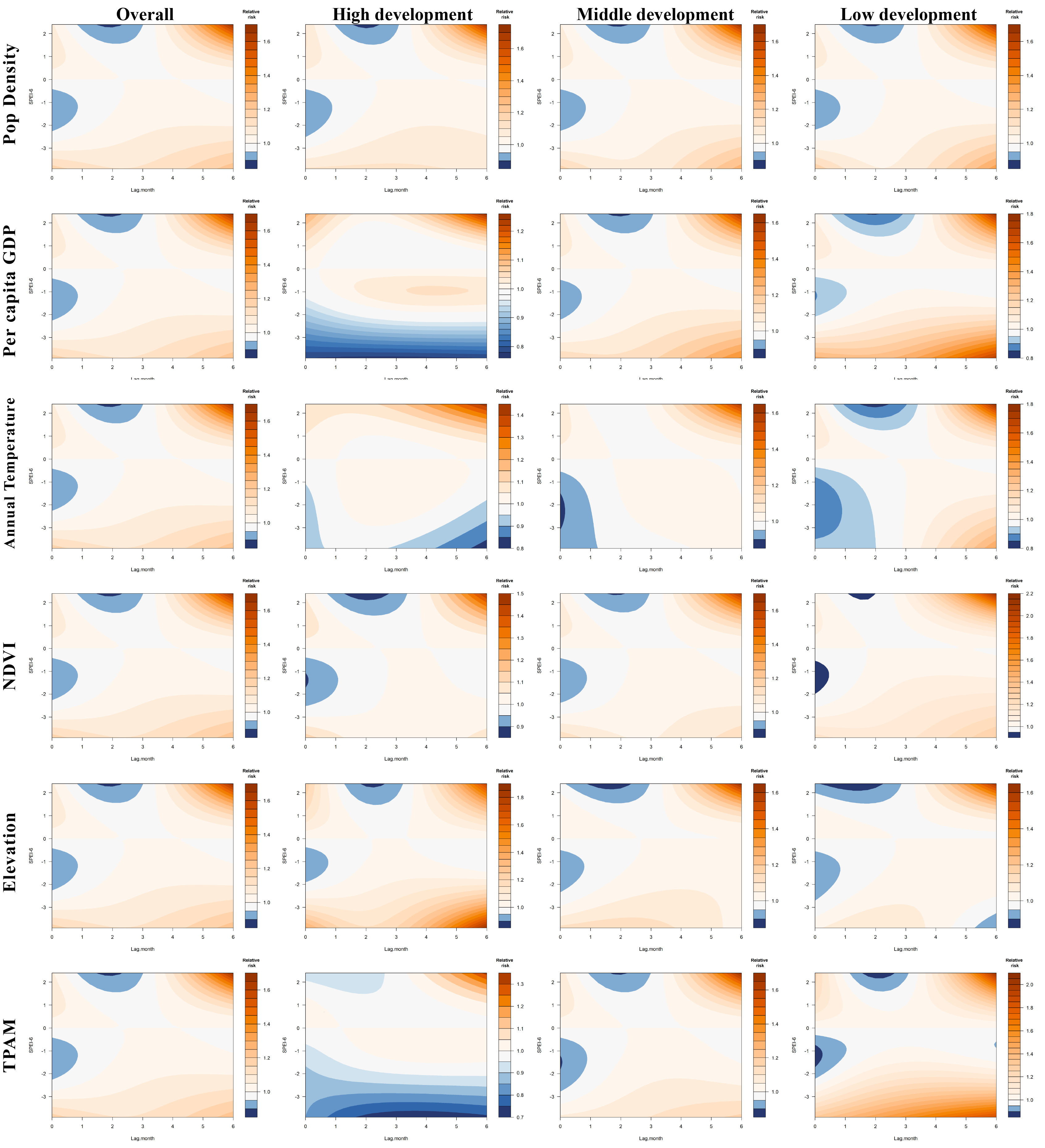

Supplement: S11 Fig — (TIF) [file pntd.0013306.s016.tif]
